# Quantification of substoichiometric modification reveals global tsRNA hypomodification, preferences for angiogenin-mediated tRNA cleavage, and idiosyncratic epitranscriptomes of human neuronal cell-lines

**DOI:** 10.1016/j.csbj.2022.12.020

**Published:** 2022-12-19

**Authors:** Florian Pichot, Marion C. Hogg, Virginie Marchand, Valérie Bourguignon, Elisabeth Jirström, Cliona Farrell, Hesham A. Gibriel, Jochen H.M. Prehn, Yuri Motorin, Mark Helm

**Affiliations:** a Institute of Pharmaceutical and Biomedical Sciences, Johannes Gutenberg University Mainz, Staudingerweg 5, 55128 Mainz, Germany; b Université de Lorraine, CNRS, INSERM, IBSLor (UAR2008/US40), Epitranscriptomics and RNA Sequencing Core Facility, F54000 Nancy, France; c Department of Physiology and Medical Physics and SFI FutureNeuro Research Centre, Royal College of Surgeons in Ireland, St. Stephen's Green, Dublin, D02 YN77, Ireland; d Université de Lorraine, CNRS, IMoPA (UMR7365), F54000 Nancy, France

**Keywords:** TRNA, Modification, TRNA fragments, Angiogenin, Modification mapping, RNAseq

## Abstract

Modification of tRNA is an integral part of the epitranscriptome with a particularly pronounced potential to generate diversity in RNA expression. Eukaryotic tRNA contains modifications in up to 20% of their nucleotides, but not all sites are always fully modified. Combinations and permutations of partially modified sites in tRNAs can generate a plethora of tRNA isoforms, termed modivariants. Here, we investigate the stoichiometry of incompletely modified sites in tRNAs from human cell lines for their information content. Using a panel of RNA modification mapping methods, we assess the stoichiometry of sites that contain the modifications 5-methylcytidine (m^5^C), 2’-O-ribose methylation (Nm), 3-methylcytidine (m^3^C), 7-methylguanosine (m^7^G), and Dihydrouridine (D). We discovered that up to 75% of sites can be incompletely modified and that the differential modification status of a cellular tRNA population holds information that allows to discriminate e.g. different cell lines. As a further aspect, we investigated potential causal connectivity between tRNA modification and its processing into tRNA fragments (tiRNAs and tRFs). Upon exposure of cultured living cells to cell-penetrating angiogenin, the modification patterns of the corresponding RNA populations was changed. Importantly, we also found that tsRNAs were significantly less modified than their parent tRNAs at numerous sites, suggesting that tsRNAs might derive chiefly from hypomodified tRNAs.

## Graphical Abstract

**Figure:**
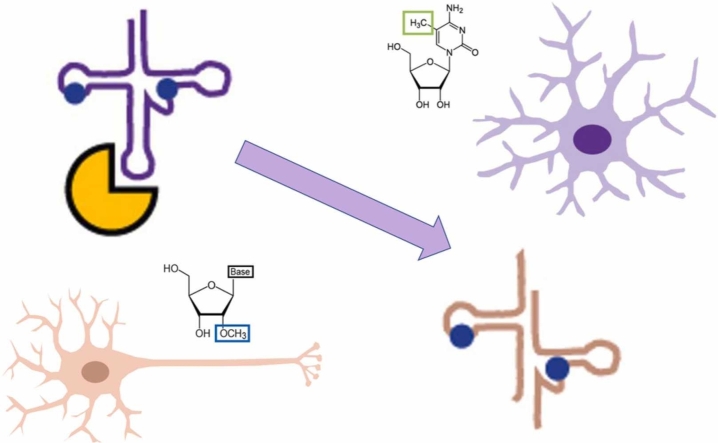

## 1 Introduction

Transfer RNA (tRNA) molecules are key players in implementation of the genetic code in the living cell. These relatively short RNAs (75–90 nt in average) are responsible for mRNA decoding by specific codon-anticodon interactions and also provide activated amino acids for protein synthesis. tRNAs are well known to contain numerous modified nucleotides, generally clustered in structurally and functionally important tRNA regions (e.g. anticodon). Due to redundancy in the genetic code, several tRNAs (called isoacceptors) may correspond to the same amino acid, encoded by different codons, moreover, tRNAs decoding the same codon may have slightly different sequence (isodecoders).

The role of tRNA and tRNA modifications in neurological disorders such as Amyotrophic lateral sclerosis (ALS), frontotemporal dementia (FTD) and Parkinson’s disease (PD), was driven to the spotlight with the demonstration of an impact of RNA modifications on tRNA processing into tRNA-derived fragments of different size, generally referred as tsRNAs, or, for miR-like fragments as tRFs [1]. Such processing is a complex process, involving a number of different nucleases, including angiogenin (ANG), with the latter cleaving tRNA in the anticodon loop and thus generating so-called tiRNAs [2], [3] while other processing steps that give rise to tiRNA-type fragments are ANG-independent [4]. For the remainder of this manuscript, we will use the term tsRNA for simplicity. A central finding in this field was that the tRNase activity of ANG, otherwise known as an important stimulator of the growth of new blood vessels, was sensitive to tRNA modifications, in particular 5-methylcytidine (m^5^C), generated by the RNA methyltransferase (MTase) activity of Dnmt2 [5] and NSUN2 [6]. tRNA cleavage in Ang-dependent and independent manners was reportedly influenced by the presence of 1-methyladenosine (m^1^A) [4], [7], subsequent work identified ribose methylations (Nm) as protective against RNase activities [8], [9]. In contrast, some tRNA modifications in the anticodon loop, like Inosine and hypermodified U* 34 may also promote accelerated tRNA cleavage by specific tRNA nucleases, like endonuclease V and zymocin, respectively [10], [11], [12], [13]. While some of the chemical structures of the modifications offer plausible biochemical mechanisms of protection against RNA cleavage [14], [15], a systematic evaluation of protective m^5^C and Nm residues is missing, as is an exploration of the effect of other modifications.

tRNAs represent 8–10% of the cellular total RNA population, making those molecules in general, as well as each individual tRNA isoacceptor, highly abundant RNA species. In general, the amount of tRNAs being processed into tsRNAs is way too low to significantly change the abundance of the cellular tRNA pool, and therefore unlikely to produce a pronounced phenotype rooted essentially in tRNA abundance. In contrast, the tsRNAs resulting from tRNA processing make up a significant fraction of the pool of small noncoding RNAs in the size range of 15–40 nucleotides or so. They have been reported to exhibit numerous downstream activities, including impact on protein biosynthesis [16]. An important finding relating this context to neurological disorders was the identification of ANG loss-of-function mutations in ALS [17]. Subsequently, ANG was shown to display neuroprotective activities [18], raising the possibility that its RNase activity might also have beneficial properties, and that RNA modification enzymes, in turn, might counter such beneficial effects. Importantly, ANG can induce tRNA cleavage upon its penetration into target cells [19], allowing a re-enactment of this scenario in tissue culture via treatment with exogenous ANG added *in cellulo*.

An open question in neuroscience is hence, which tRNA modifications modulate tRNA processing into tsRNAs in general, and by ANG in particular [20], [21], [22]. We have recently shown, by quantitative LC-MS of tRNA isolates from different brain tissues, that the tRNA modification levels of cerebellum hippocampus and cortex are sufficiently distinct to discriminate these tissues [23]. This shows that the task of identifying ANG-modulating modifications requires analysis of defined tRNA populations of pure tissue or of isolated cell lines. A further consideration is that a mere quantification of the modification content of tRNA preparations from nerve cells, as e.g. by LC-MS, does not provide any sequence context or position of modifications. Based on the above findings concerning the protective effects of m^5^C and Nm, knowledge not only of the amount, but of the specific positions of ANG-mediating modifications is highly desirable. A promising approach to this goal resides in a number of high throughput sequencing techniques, which have been recently developed to map positions of certain modifications (reviewed in [24], [25]). These methods typically make use of specific chemical, biochemical, or biophysical properties of the corresponding modification type [26]. Among these are methods tailored to the mapping of m^5^C [27], Nm [28], [29], as well as for the simultaneous mapping of 7-methylguanosine (m^7^G), 3-methylcytidine (m^3^C), and dihydrouridine (D) [30], that were applied here.

For reasons laid out above, we chose to assess ANG-modulating modifications in two tumor cell lines derived from different cellular backgrounds, namely from glial and nerve cells. Modification profile differences between these two populations will also be discussed.

## 2 Material and methods

### 2.1 Cell culture and angiogenin treatment

Human glioblastoma MZ-294 cells (RRID: CVCL_M410) derived from a primary glioblastoma [31] were grown as a monolayer in DMEM (Lonza) supplemented with 10% heat-inactivated fetal bovine serum (FBS; Sigma), 2 mM L-Glutamine (Sigma), 100 U/ml Penicillin and 0.1 mg/ml Streptomycin (P/S: Sigma) and maintained in a humidified incubator at 37 °C and 5% CO2.

The SH-SY5Y cell line (RRID: CVCL_0019), is a cloned subline of the human neuroblastoma cell line SK-N-SH derived from a metastatic bone tumour [53]. SH-SY5Y cells were obtained from the American Type Culture Collection (ATCC, CRL-2266) and grown as a monolayer in DMEM/F12 (Gibco) supplemented as above and maintained in a humidified incubator at 37 °C and 5% CO2.

Both cell lines were confirmed to be mycoplasma-free.

Lyophilized human recombinant ANG (rhANG; R&D Systems, #265-AN/CF) was reconstituted at 10 μg/ml in sterile PBS containing 1% BSA and stored at − 20 °C until use. MZ-294 cells and SH-SY5Y cells were treated with either rhANG (500 ng/ml) or vehicle (PBS with 1% BSA) in serum-free Neurobasal plus medium (Gibco) for 3 h in a humidified incubator at 37 °C and 5% CO2.

### 2.2 Immunocytochemistry

To assess ANG cellular localisation, immunocytochemistry (ICC) was performed on MZ-294 cells and SH-SY5Y treated with rhANG or vehicle. For ICC, MZ-294 cells and SH-SY5Y cells were seeded on glass coverslips in standard 24-well plates at densities of 2.5 × 10^4^ cells/well and 1 × 10^5^ cells/well, respectively. 24 h following seeding, the cells were treated with rhANG or vehicle and then subjected to standard ICC techniques. Briefly, cells were fixed in 4% paraformaldehyde for 15 min at RT, permeabilized in ice-cold 95% ethanol with 5% glacial acetic acid for 10 min at RT and blocked in 5% BSA/PBS with 0.1% Triton X-100 for 1 h at RT. Cells were incubated with the following antibodies in blocking buffer at 4 °C overnight: goat anti-human ANG (1:100, PC317L, Calbiochem, RRID: AB_213593) and mouse anti-tubulin (1:500, T6199, Sigma, RRID: AB_477583). Following primary antibody incubation, cells were incubated with the appropriate Alexa-dye conjugated secondary antibodies (1:500, Invitrogen) and Hoechst nuclear counterstain (1 μg/ml, Invitrogen) for 2 h at RT. Coverslips were mounted on glass microscope slides (Thermo Scientific) using ProLong Gold Antifade Mountant with DAPI (Invitrogen). Images were acquired using a confocal Zeiss LSM 710 microscope with a 40X oil immersion objective (EC Plan-Neofluar 40x/1.30 Oil DIC M27, Zeiss) and processed using ImageJ (National Institutes of Health, USA).

### 2.3 Total RNA extraction and tRNA/tsRNAs purification

Total RNA was extracted and processed into tRNA/tsRNAs fractions as described in Richter et al. [23]. In brief, separation of tRNA and tsRNA fragments was achieved by 10% PAGE and bands, corresponding to tRNAs (60–75 nt) and tsRNAs (∼15–50 nt) were excised and eluted using the ZR small-RNA™ PAGE Recovery Kit according to the manufacturers specifications.

### 2.4 Library preparation for tRNAs and tsRNAs fractions

Gel-extracted tRNA and tsRNAs fractions were quantified and ∼150 ng of input RNA material was directly used for specific chemical treatment, allowing detection of Nm (RiboMethSeq), m5C (BisulfiteSeq) and m7G/m3C/D residues (AlkAnilineSeq), see below for description of those methods. In all cases chemical and enzymatic treatment used was aimed to create RNA fragments having 5’-P and 3’-OH extremities. Following steps in library preparation included the use of commercial NEB Small RNA kit for Illumine, comprising ligation of adapters at the 3’-end of RNA, annealing of the RT primer, ligation et the 5’-extremity and cDNA synthesis by RT. cDNA was amplified and barcoded for Illumina sequencing.

### 2.5 tRNA reference for alignment

Construction of reduced tRNA reference dataset for alignment was described previously [32]. In brief, predicted tRNA genes from gtRNAdb database (http://gtrnadb.ucsc.edu/) were clustered based on Euclidean distance between tRNA sequences. From this clustering, consensus sequences representative of each of the gene were compiled into a single reference sequence used for reads’ alignment.

### 2.6 Sequencing detection methods

All tRNAs and tsRNAs samples have been sequenced with the HiSeq 1000 system from Illumina. Sequenced reads have been demultiplexed with bcl2fastq software, trimmed with Trimmomatic version 0.32, and aligned with bowtie2 version 2.2.2 on a Linux Server Power Edge R815 from Dell. Their subsequent analyses from this point depends on the method used (RiboMethSeq, AlkAnilineSeq and Bisulfite Sequencing) on R software version “Bird Hippie” 4.1.2 with RStudio server integrated development environment (IDE) version “Juliet Rose” 1.4.1717. Data treatment and scores calculations have been done through R basic functions, heatmaps have been created from the “heatmap.2” function from the *gplot* package and the other graphs have been designed through the *ggplot2* package.

The demultiplexed unprocessed reads are available on ENA platform with accession number PRJEB57983.

### 2.7 RiboMethSeq

RiboMethSeq protocol has already been described in detail previously [29], [33], [34]. RiboMethSeq is based on alkaline hydrolysis protection resulting from 2’-O-methylation. This modification located in the vicinity of the phosphodiester bond of RNA molecules prevents the deprotonation of the 2’-OH moiety which can interact with the neighboring phosphate group resulting in a 2’− 3’-cyclic phosphate formation occurring a RNA cleavage. An alkaline treatment on RNA sequences of interest allows the creation of a cleavage profile created from 5’ and 3’ reads’ extremities counting through sequencing data manipulation in order to analyze such profile for cleavage protection detection. The detection and quantification of this alkaline protection is done through analysis of RiboMethSeq Scores, namely Score A (also known as Score A2), Score C (also known as Score C2 or MethScore), Score mean (or mean2) and Score Angle. Score C is used as a quantitative score for stoichiometry assessment. Here is the different formula for the RiboMethScores:

$$\mathrm{Score}A=1-\frac{2n_{i}+1}{\frac{1}{2}\left|\mu_{l}-\sigma_{l}\right|+n_{i}+\frac{1}{2}\left|\mu_{r}-\sigma_{r}\right|+1}$$

with n_i_, the number of 5’/3’-end reads counts at given position i.

μ_l_ and μ_r_, the 5’/3’-end reads’ counts average from the preceding (left region) or subsequent (right region) neighboring nucleotides of position i, respectively. The range of these regions is 2 positions in this study.

$$S\mathrm{core}B=\frac{n_{i}-\frac{1}{2}\left(\frac{\sum_{j=i-\delta }^{i-1}\omega_{j}n_{j}}{\sum_{j=i-\delta }^{i-1}\omega_{j}}+\frac{\sum_{j=i+1}^{i+\delta }\omega_{j}n_{j}}{\sum_{j=i+1}^{i+\delta }\omega_{j}}\right)}{n_{i}+1}$$

The same method applies for σl and σr, which are respectively the 5’/3’-end reads’ counts standard deviation of the left and right region.

$$\mathrm{Score}C=\frac{n_{i}}{\frac{1}{2}\left(\frac{\sum_{j=i-\delta }^{i-1}\omega_{j}n_{j}}{\sum_{j=i-\delta }^{i-1}\omega_{j}}+\frac{\sum_{j=i+1}^{i+\delta }\omega_{j}n_{j}}{\sum_{j=i+1}^{i-\delta }\omega_{j}}\right)}$$

with n_i_ and n_j_, the number of 5’/3’-end reads counts at given position i and j, respectively.

δ is the range of the neighboring positions taken in account (2 in this study).

ω_j_ is the incremented weight attributed to each neighboring positions, ranging from 1 when j = i + /- 1 (subsequent positions) to 0.5 when j = i + /- 6. Further is j from i, lower is the weight.

$$r_{i}=\frac{n_{i}}{n_{i-1}}f_{i}=\frac{n_{i}}{n_{i+1}}$$

$$\mathrm{ScoreMEAN}=\frac{\left(1-\frac{r_{i}}{\frac{\sum_{j=i-\delta }^{i-1}r_{j}}{\delta }+\frac{\sum_{j=i+1}^{i+\delta }r_{j}}{\delta }}\right)+\left(1-\frac{f_{i}}{\frac{\sum_{j=i-\delta }^{i-1}f_{j}}{\delta }+\frac{\sum_{j=i+1}^{i+\delta }f_{j}}{\delta }}\right)}{2}$$

with n_i_, the number of 5’/3’-end reads counts at given position I and δ the range of the neighboring positions taken in account (2 in this study).

### 2.8 AlkAnilineSeq

As described in previous publications [30], [35], the AlkAnilineSeq method for the detection of m^7^G is based on RNA mild alkaline hydrolysisleading to a ring opening of m^7^G and form an abasic site which can be treated by aniline to create a 5’-P suitable for detection by high-throughput sequencing. The method was found to also react to the presence of other modifications such as m^3^C and D, presumably leading to an abasic site as a common denominator. One important aspect of this method is the selection of RNA abasic sites through the direct adapter ligation to the 5’-phosphate generated by aniline treatment. The main score used for m^7^G detection is Normalized Cleavage score but this score is not suitable for tRNA analyses. Therefore, stop ratio, a ratio between the number of reads’ 5’-extremities occurring at a position of interest (pinpointing direct 5’-adapter ligation due to the presence of an abasic site) and the total number of reads passing through the same position gives insight as a detection of quantification of m^7^G sites.

$\mathrm{stop}=\frac{\mathrm{Reads starts}}{\mathrm{Total number of reads}}$

### 2.9 Bisulfite sequencing

Bisulfite sequencing procedure has been described in numerous papers in the literature [27], [36]. This method is based on the deamination of cytidine by the bisulfite ion (HSO_3_^-^) which results in the formation of a uridine. However, the methyl group of m^5^C prevents this deamination to occur, allowing a distinction between unmodified cytidine converted to uridine and m^5^C which remains unconverted cytidine. In order to pinpoint m^5^C sites, a bisulfite non-conversion rate (NonConv) is calculated. It is a ratio which is calculated for each C residue and represents the proportion of reads harboring a C at this given position and the cumulated numbers of reads harboring either a C or a U. Since m^5^C is not converted to uridine, the number of reads having C at those positions can be served as a detection tool but also as a quantitative score since this rate is directly proportional to m^5^C presence.

$\mathrm{NonConv}=\frac{\mathrm{Unconverted cytidines}}{\mathrm{Cytidines converted to uracils}+\mathrm{Unconverted cytidines}}$

### 2.10 Principal component analysis (PCA)

All PCAs analyses has been conducted through the function *prcomp()* from the R package *stats* using the correlation table of our data containing the median of 3 samples of quantitative score respective of each methods; Bisulfite non-conversion rate for BisulfiteSeq, Score C for RiboMethSeq and stop ratio for AlkAnilineSeq. All m^7^G sites due to their constant high stoichiometry have been excluded prior to analysis for noise reduction.

## 3 Results

Our previous demonstration that different brain tissues can be distinguished by their tRNA modification patterns [23] led us to investigate if such was also the case for human brain-derived cell lines. We therefore isolated tRNA and small RNA fragments from glioblastoma cell line MZ-294 (MZ) and neuroblastoma cell line SH-SY5Y (SH) (see Material and methods). Total RNA was extracted from cultured cells and size-fractionated by denaturing PAGE. Bands corresponding in size to tRNAs and tsRNAs, respectively, were excised and eluted using standard methods described previously [23].

### 3.1 Mapping of known and new putative m^5^C, 2’O-methylated (Nm), m^3^C, D and m^7^G sites in human tRNAs

While our previous investigations assessed the overall modification content of tRNA fraction and small RNA fragments by LC-MS [23], we here chose a more sophisticated approach, namely pinpointing the location of tRNA modifications by RNA-seq based methods specifically tailored to mapping of (i) m^5^C residues by bisulfite sequencing [27], (ii) ribose-methylated nucleotides Nm by RiboMethSeq [33], and (iii) alkali-sensitive modifications D, m^3^C, and m^7^G, by AlkAnilineSeq [30]. In all three methods, reads were aligned to a synthetic “tRNA genome”, the tRNA reference sequences specifically designed for this type of analysis [32]. This reduced tRNA genome accounts for the occurrence of 39 cytoplasmic tRNA sequence families, each one consolidating sequences of high similarity, such as isoacceptors/isodecoders that differ only by a few SNPs. Selection of putative modified sites was based on threshold application on “detection scores”, which were developed along with the methodology itself [30], [37], [38].

#### 3.1.1 General considerations

For all three methods, we first performed an “unbiased” evaluation of our current data according to threshold values and considerations on noise, whose details are developed below. An overarching consideration of particular importance is rooted in the objective, which differs from many previous studies. Importantly, our objective was not to detect as many new modification sites as possible by setting low detection thresholds, but rather to assess variations of sub-stoichiometrically modified sites. Specifically, we wanted to detect variations of modifications between two human cell lines, which can only be achieved at tRNA sites that are incompletely modified, and therefore remain plausible substrates for modification enzymes. We posit that, for this to be relevant, a signal above noise level would have to be present in at least half of the tRNA samples investigated. Therefore, a criterion for our positive list of to-be investigated sites was occurrence of a signal in at least 50% of the biological samples. Furthermore, to be able to quantify the extent of substoichiometric modification, sites with low read coverage (below 30 reads per position) were not considered.

In a further selection step for appropriate modification sites, we applied plausibility considerations based on known RNA modification enzyme specificities, as well as known tRNA modification sites reported in the literature. This entailed removal of candidates at positions within the tRNA sequence and structure, which had not been reported as substrate of enzymes conferring the particular modification under scrutiny. For example, m^5^C was never found at positions 11, 12, or 17 in tRNA, nor was any corresponding enzymatic activity in any organism reported in the literature. Residual non-deaminated C residues at those locations likely result from lower chemical reactivity to bisulfite due to local RNA structure, rather than from real presence of m^5^C. Corresponding candidates were therefore deemed implausible and discarded. Inversely, modifications established by published experimental evidence, which our unbiased assessment had failed to detect based on the scoring system, were added back in. One example detailed below concerns Gm18, a well-established modification in the D-loop of several human tRNAs, which fell through our scoring system (vide infra), potentially due to substoichiometric modification.

#### 3.1.2 Bisulfite mapping of m^5^C residues

RNA bisulfite sequencing is derived from well-established DNA bisulfite sequencing and is based on conversion of all C residues to U upon chemical deamination. A subset of modified C residues, e.g. m^5^C, m^4^C are resistant to deamination and can be detected by deep sequencing as non-converted C residues. In bisulfite sequencing, cytidines were considered modified when their signal, i.e*.* the non-conversion rate (NonConv) was at least 3 times the random non-deamination noise, a widespread custom in analytical chemistry [39], [40]. To assess this noise, NonConv distribution on reads aligned to rRNAs from the same samples was analyzed. The NonConv background from these *bona fide* unmodified cytidines (all rRNA C sites except 2 known m^5^C positions in 28 S rRNA) showed an average of 0.0252 and a standard deviation of 0.0677 resulting in a threshold value set to 0.228 (average + 3 *standard deviation) (see Fig. S1).

**Fig. 1:**
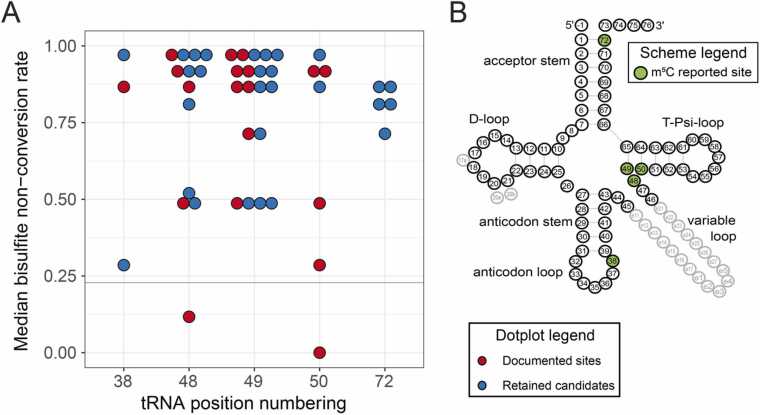
BisulfiteSeq mapping of m^5^C sites in tRNAs. m^5^C modification level was measured here as bisulfite NonConversion level C-to-U (NonConv) and plotted on the Y-axis for tRNA positions (x-axis) reported to be modified in at least one isoacceptor in any species. The threshold of average + 3 *standard deviation = 0.228 is highlighted by a green horizontal line. Each dot represents a site in a different tRNA sequence. The red dots represent the m^5^C sites known from the literature [[45], [46], blue dots are plausible additional m^5^C sites identified in other tRNAs according to their NonConv score. The cloverleaf to the right shows the canonical secondary tRNA structure along with tRNA-specific nucleotide position numbering, including extra (optional) residues 17a, 20a/20b in the D-loop as well as nucleotides e1-e27 in the variable tRNA loop/region. Positions relevant for m^5^C are shown as green dots (pos 38, 48_49_50 and 72).

Hence the threshold is well in line with standard bisulfite sequencing analyses in the literature [41], [42]. In keeping with the above working hypothesis for analyzing fractional occupancy, i.e*.* substoichiometric modification of a given site in different biological contexts, we pursued our analysis only for those C sites which exceed the threshold NonConv > 0.228 in at least half of the analyzed tRNAs samples. Furthermore, the plausibility of an m^5^C modification site was assessed based on known specificities of m^5^C-MTases, To this end, each site was localized according to the tRNA numbering system [43] in order to homogenize site attribution. 4 sites, namely (Ser(GCT)11, Glu(CTC/TTC)12, Leu(TAA)12 and Lys(CTT)17) were excluded, since their localization did not correspond to any known m^5^C modification site or tRNA:m^5^C-MTase targeted sites [44]. The latter included positions 38 (modified by DNMT2); 48, 49 and 50 (NSUN2) and 72 (NSUN6). Fig. 1 shows the final 47 curated sites (including 2 referenced ones which have not been seen through our detection parameters) in ascending order of tRNA numbering on the x-axis, and a plot of NonConv median values on the Y-axis, where the threshold of 0.228 is highlighted by a green horizontal line. The red dots represent the m^5^C tRNA sites known from the literature “documented sites” [45], [46] while the blue ones are the positions identified as highly plausible additional m^5^C sites (“retained candidates”).

The above findings indicate that the identified modification sites are highly plausible, and that the threshold used here is meaningful for sensitive and specific m^5^C detection by bisulfite sequencing, even at sub-stoichiometric modification level.

#### 3.1.3 RiboMethSeq mapping of Nm residues

**Fig. 2:**
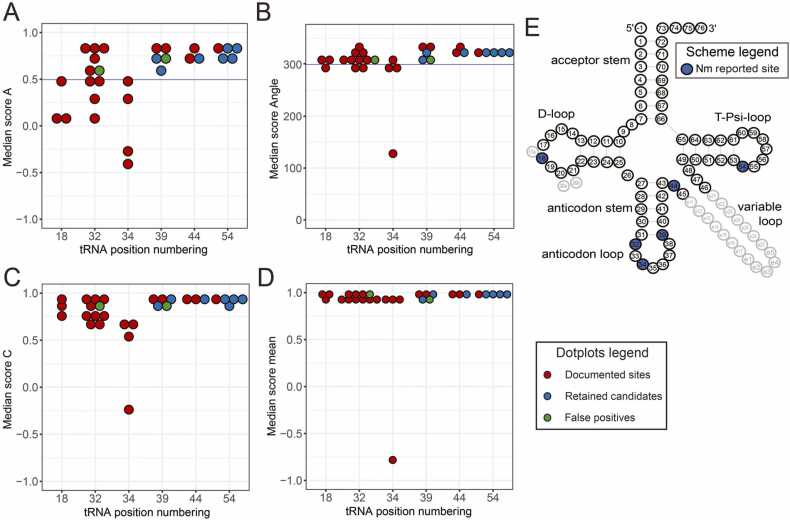
RiboMethSeq mapping of Nm sites in tRNAs. The upper panels A and B show results according to Score A and Score Angle, both of which were used for selection based on thresholds (blue lines). The lower panels show plots of Score mean and Score C, which were not applied for selection, but allow multiple types of assessment for any given residue. Red dots indicate known modification sites from the literature. Green dots indicate positions featuring high RiboMethSeq scores, despite being described as unmodified in the literature. These were therefore discarded. Blue dots indicate positions with scores above threshold values that represent plausible Nm modification sites. The cloverleaf to the right shows the canonical secondary tRNA structure along with tRNA-specific nucleotide position numbering, including extra (optional) residues 17a, 20a/20b in the D-loop as well as nucleotides e1-e27 in the variable tRNA loop/region. Positions relevant for Nm modification are shown as blue dots (pos 18, 32, 34, 39, 44 and 54).

The RiboMethSeq protocol, developed for the detection of Nm residues in RNA assesses protection of RNA phosphodiester bonds against alkaline hydrolysis. In 2’-O-methylated residues, the 2’-OH nucleophile is capped by a methyl (-CH_3_) group, thus abolishing cleavage of the 3’-adjacent phosphodiester bond almost completely. Therein RiboMethSeq analysis faces noise from unrelated, non-alkaline RNA hydrolysis on one hand, and spurious protection against hydrolysis resulting from factors unrelated to modification, e.g., RNA secondary and tertiary structure. Other biases, namely at the adapter ligation steps are also known to affect the RiboMethSeq protection profile. Different score types developed for this protocol are applied to an ensemble of reads, without possibility to draw conclusion from a single-molecule digital conversion versus non-conversion event, as is the case for BisulfiteSeq. For several scores developed in previous publications, all signals were contained within three standard deviations of the noise, meaning that we needed to develop other threshold values. An analysis showed that the distribution of values Score A followed a Gaussian distribution, and the same applied to Score Angel (See Fig. S2). Because Score A is rather specific, while Score Angle is sensitive, we decided to work with Nm candidates that were identified by both scores. In keeping with a conservative approach, for each of both scores, candidate sites were retained that scored above the mean noise plus 2 standard deviation, thus excluding 97.7% of noise and correspondingly accepting the remaining 2.3% as a baseline for false positives. The overlap of candidates from both scores was then scrutinized for biological plausibility according to known enzymatic activities that catalyzed the formation of Nm residues in tRNA. For reasons explained above, we added back-in candidates, such as Gm18 and U*m34 (U* stands for various wobble modifications), for which published experimental evidence was available, despite their scores being below noise + 2 * standard deviation in one (or both) of the scores. An exception concerns Nm residues at position 4, whose placement near the 5’-end of tRNA prevents reliable quantification by RiboMethSeq. The results shown in Fig. 2 feature the Score A and Score Angle on the Y-axis in panels A and B, respectively. The threshold values are indicated by a blue line, and dots are color-coded with respect to their validation from literature (in red) and meeting the threshold minimum. The latter are divided into two categories, namely plausible sites (in blue) and false positives, known from literature to be unmodified or carrying another known modification comprising no ribose methylation.

Note that, as a consequence of its specificity, a relatively large number of literature-known sites (red color) candidates are below threshold in Score A. These include positions 32 and 34, whose signals are frequently part of highly irregular RiboMethSeq protection profiles in the tRNA anticodon loop, in part due to hypermodified (and also RT-arresting) residues at position 37. Panels C and D show two additional scores, including score “mean”, which is another sensitive detection score such as Score Angle. The bottom right panel shows Score C, which is linear with respect to modification stoichiometry, and therefore the score of choice for quantification in what follows (v*ide infra*). Further validation of Gm18 and Nm32/34 sites was obtained by independent analysis of datasets for respective human KO cell lines [8], [28]. In summary, 29 sites were retained from this analysis, which we quantitatively analyzed to characterize differences between the two cell lines (vide infra).

#### 3.1.4 AlkAnilineSeq mapping of m^7^G, m^3^C, and D residues

A subset of RNA modifications was shown to be sensitive to the combined action of heat and of alkaline pH, leading to scission of the N-glycosidic bond with subsequent formation of an RNA abasic site. Aniline treatment opens the RNA abasic site, inducing cleavage of the RNA phosphodiester bond, and thereby releasing 5’-phosphate-containing RNA fragments. Because the 5’-phosphates present a unique “molecular entry” to library preparation, the AlkAnilineSeq protocol features very low noise levels. For each modification and, thus, the parent nucleotide, the background is different, and signal strength differs between the different modifications.

**Fig. 3:**
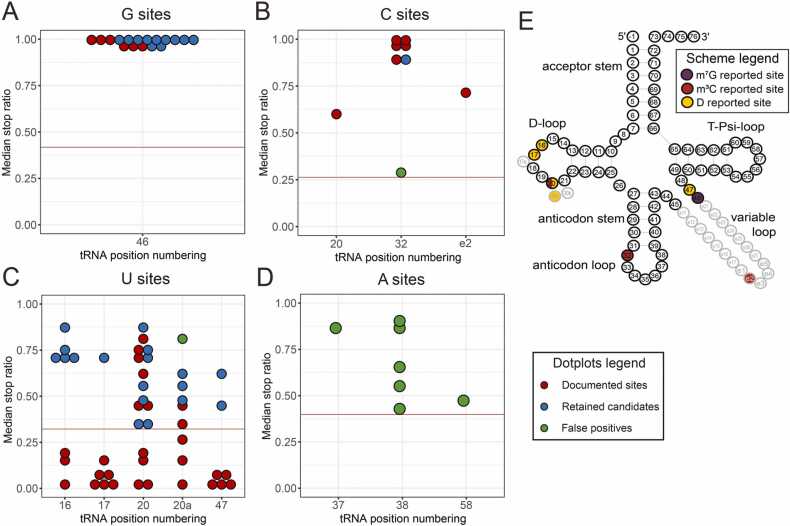
AlkAnilineSeq mapping of m^7^G, m^3^C, and D sites in tRNAs. Panels A-D show plots of stop ratio scores with corresponding thresholds indicated by horizontal pink lines. Red and green dots indicate, known modified and unmodified sites, respectively, from the literature. Known unmodified sites were therefore discarded. Blue dots indicate sites having scores above threshold values that represent plausible m7G/m3C/D modification sites. The cloverleaf in panel E shows the canonical secondary tRNA structure along with tRNA-specific nucleotide position numbering, including extra (optional) residues 17a, 20a/20b in the D-loop as well as nucleotides e1-e27 in the variable tRNA loop/region. Positions relevant to m7G-modification violet dots (pos 46), m3C sites are in red (pos 20, 32 and e2), D sites are in yellow (pos 16, 17, 20, 20a and 47).

The score applied for mapping of all nucleotide modifications was median stop ratio, with thresholds defined as three times the standard deviation (Fig. S3) ranging from 0.263 to 0.418, as indicated by horizontal red lines in Fig. 3. The color code of the candidates conforms to that used in previous Fig. 1, Fig. 2. Red dots label sites that were already reported, blue stand for sites that are plausible, but so far unknown, and green denote sites known to be unmodified, or containing other types of modification, respectively.

The strongest signals in AlkAnilineSeq originated from m^7^G residues, whose signals were all close to the maximum value of 1 (Fig. 3 A) with a threshold of 0.418. All candidates derive from the only known m^7^G modification site in tRNA at position 46. Signals derived from m^3^C sites as shown in Fig. 3B, were generally well separated from noise, with one exception. Said exception concerns position 32 in tRNA^SeC^ (TCA) (Fig. 3B), green dot just above 0.26 threshold line. According to the Modomics database, this C32 is not modified,and therefore was discarded. Known m^3^C sites include positions 32 and position e2 in the variable loop in several tRNAs, as well as one reported m^3^C at position 20 [47]. Two AlkAnilineSeq signals at position 26 for tRNA^Trp^(CCA) and tRNA^Tyr^(GUA) could not be plausibly assigned. (supplement "Supp_Table_all_discarded_candidates.csv"). Both these tRNAs feature a guanosine at the relevant position 26, where the only known natural tRNA modification is m^22^G, (rather than m^7^G). The chemical properties of this doubly methylated nucleotide are not compatible with cleavage induced under conditions of the AlkAnilineSeq protocol, and other tRNAs with known m^22^G26 do not show such a signal. Since, in addition, these sites are not a known target of m^7^G-MTases, they were discarded. Since the AlkAnilineSeq protocol is intrinsically sensitive to RNA abasic sites, these unexpected G signals may be related to spontaneous loss of purine residues in RNA.

For D residues, which are uridine derivatives, the stop ratio noise average was 0.0329 with a standard deviation of 0.0964, setting the threshold for D sites at 0.322 (Fig. 3C). At typical tRNA D-sites at positions 16, 17, 20 and 20a and 47, known to be targeted by Dus enzymes, the signal strength was rather variable. As shown by red dots in Fig. 3C, many known sites of modification showed signals well below our detection threshold. This might plausibly be explained by substoichiometric modification, which is a hallmark of dihydrouridine [48]. One 20a site from tRNA^Asn^(GTT) was also discarded since this position was reported to be modified to acp^3^U.

The adenosine nucleosides serve as a control, since there are no known chemical structures of adenosine derivatives that respond to the chemistry of the AlkAnilineSeq protocol. With a noise average of 0.0473 and a standard deviation of 0.117, the stop ratio threshold for A sites is 0.398, which is rather high in comparison to the other nucleosides. Interestingly, there are a number of signals observed above threshold (Fig. 3D). However, all but one occur at, or next to, known bulky anticodon modifications, which are thought to interfere with an adapter ligation step in the library preparation protocol.

#### 3.1.5 A selection of tRNA modification sites for assessment of stimulus-dependent modification dynamics

**Fig. 4:**
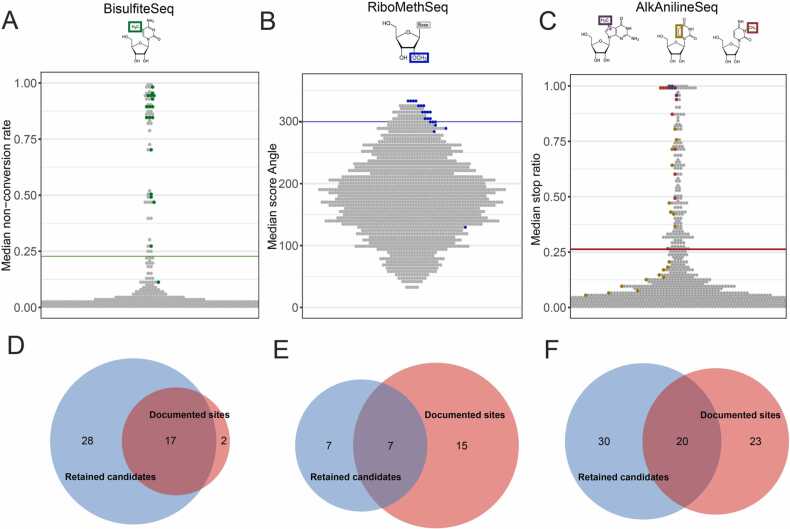
Experimentally mapped sites for m^5^C, 2’-O-methylation and m^7^G/m^3^C/D compared to data from the literature. (A-C) Relevance of applied threshold for detection of putatively modified sites (each dot represents one site) compared to known modified sites represented by green dots for BisulfiteSeq (A), blue dots for RiboMethSeq (B) and purple (m^7^G), gold (D) and orange (m^3^C) dots for AlkAnilineSeq (C). The respective thresholds are shown by colored horizontal lines: 0.228 for bisulfite non-conversion rate, 300 for the score Angle and 0.263 for stop ratio. Values correspond to score medians from 12 tRNA samples, 3 untreated and 3 treated with angiogenin for both cell lines. (D - F) Overlapping and exclusivity between tRNA sites known from the literature (“documented sites”, red circle) and tRNA sites detected through our workflow (“retained candidates”, blue circle). Panels represent data from BisulfiteSeq (D), RiboMethSeq (E) and AlkAnilineSeq (F), respectively.

In our objective to find tRNA modification sites suitable to gauge the impact of physiological conditions such as cell type and exposure to angiogenin, the above workflow combined with data from literature identified 149 positions in human tRNAs as being robustly modified. While there are certainly additional sites to be found, those summarized in Fig. 4 and Table S1 are amenable to our analytics. Sites include 47 m^5^C, 29 Nm, 47 D, 9 m^3^C, and 17 m^7^G modifications (summarized in Table S1 and Fig. S4). As shown in Fig. 4A-C, the dynamic range of the different scoring systems is relatively heterogeneous. Still, summarized over the different scoring in Figs. 1–4, a total of 35 of these modification sites apparently feature complete modification stoichiometry across tRNAs and tsRNAs (> 80% estimated modification level) with 10 m^5^C, 7 Nm, 1 m^3^C and all 17 m^7^G sites. All 149 sites might change their modification status, but especially the substoichiometrically modified sites are prime candidates for comparative analysis of tRNA modification profiles, most liable to change said stoichiometry under alternate cellular physiology.

### 3.2 Modification patterns of tRNA versus tsRNAs show significant differences

**Fig. 5:**
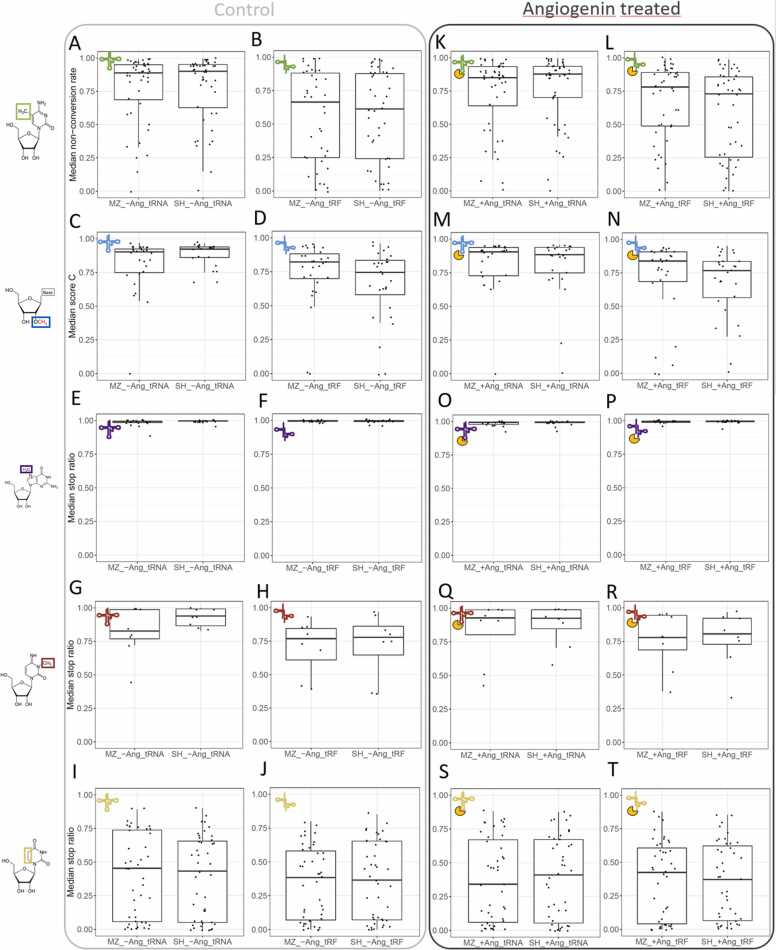
Box plots showing modification levels for tRNAs and tsRNAs extracted from untreated and ANG-treated samples. Panels (A-J) correspond to untreated samples, while panels (K-T) show the results for ANG-treated cells. Modification levels for m^5^C (A, B, K, L), Nm (2’-O-Me) (C, D, M, N), m^7^G (E, F, O, P), m^3^C (G, H, Q, R) and dihydrouridine D (I, J, S, T) are shown. The values shown here are modification level medians of the respective detection scores obtained for three biological replicates.

From the above list of modifications deemed amenable to robust quantification, an assessment of their modification stoichiometry was done in biological triplicate. This analysis was extended from full length tRNA to tRNA fragments. The results are shown as boxplots in Fig. 5, with separate plots for each modification. Plots in the first column (panels A,C, E,G) show tRNA modification profiles of the MZ and SH cell lines, whereas plots in the second column (B,D,F,H, Note that an alternative arrangement of the panels is available in the supplement in Fig. S5) show the corresponding results obtained for respective tsRNAs. Results for p-values obtained from a comprehensive comparison according to Mann–Whitney–Wilcoxon test [49] are given in Tables S2-S6 in the Supplement. Differences between tRNAs of the two cell lines used were observed for m^3^C and m^7^G levels. More significant differences (p-value < 0.01) between tRNAs and tsRNAs were found for m^5^C and Nm, and significant differences (p-value threshold of 0.05) also exist for m^3^C. Globally, the tsRNAs appear less modified compared to intact tRNAs.

### 3.3 Treatment with angiogenin *in cellulo* induces moderate changes in the modification patterns of tRNAs and tsRNAs

**Fig. 6:**
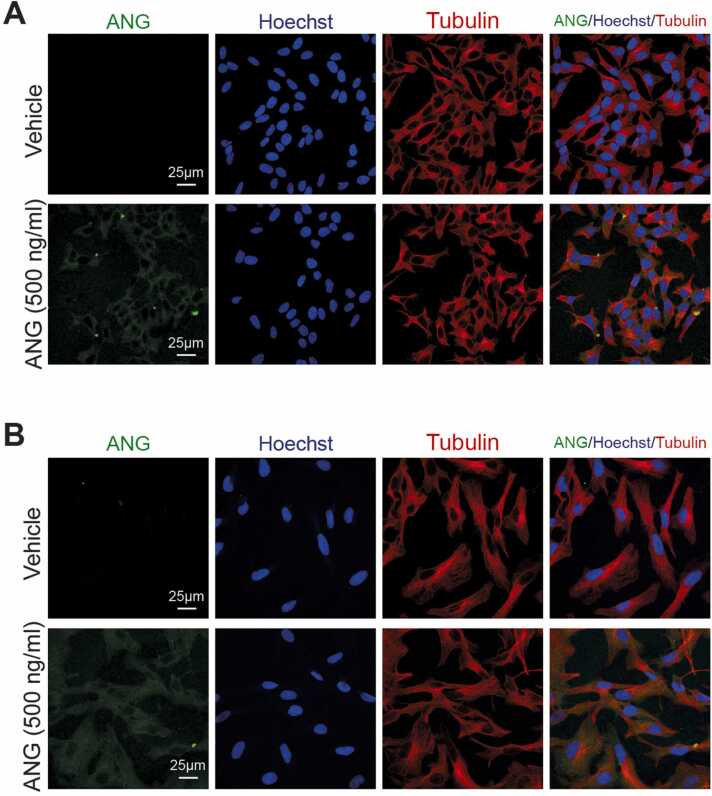
Uptake and subcellular localization of ANG in MZ and SH cell lines. (A-B) Confocal images of the intracellular distribution of ANG in (A) SH-SY5Y cells and (B) MZ-294 cells following 3 h treatment with recombinant human ANG (500 ng/ml) or vehicle. The cytoplasm is stained with tubulin (red) and nucleus is stained with Hoechst (blue). Scale bars, 25 µm. Images are representative of n = 3 experiments.

Because ANG has emerged as an important player in the processing of tRNAs into tsRNAs on one hand, and in CNS related diseases on the other hand, we decided to test its impact on tRNA/tsRNA modification patterns *in cellulo*. This was made possible by ANG’s capacity to penetrate cells, documented by several groups [19], [50], [51]. Following a previously published regimen [52], both cell lines were treated with ANG, and ANG uptake was verified by fluorescence microscopy as detailed in the Materials and Methods section. Fig. 6 shows the intracellular distribution of ANG (green stain, left column) being mainly cytosolic at the time of analysis, while entering the nucleus at a later time point [52]. Activity of Angiogenin has been verified by evaluation of RNA fragmentation in MZ and SH cells (Fig. S6).

After treatment, cells were harvested, and RNAs were isolated and analyzed as described above. Panels K-R in Fig. 5 show the corresponding data in boxplots, with tRNA data in the third column (panels K,M,O,Q,S), and tsRNAs data in the fourth column (panels L,N,P,R,T. Note that an alternative arrangement of the panels is available in the supplement: Fig. S5). Overall, when perusing the ensemble of tRNA modification sites, angiogenin did have only mild effects, which can be appreciated by comparing the boxplots in Fig. 5 in a given left panel with the associated plot in the right panel. While shifts of the median value and changes in standard deviations occur in several instances, none of the angiogenin-induced changes are significant with respect to the ensemble of sites. For example, m^5^C value plots tend to display lower dispersion after ANG treatment (compare Fig. 5A with 5 K, and 5B with 5 L). No general direction could be identified by boxplots for Nm, m^7^G, m^3^C and D (Fig. 5C-J, and Fig. 5M-5 T). As above, the p-values in tables S1-S5 indicate significant differences between modification levels of tRNAs and tsRNAs as a general rule, but no other significant differences are in evidence. Since the RNase activity of ANG is known to be impeded by selected tRNA modifications, this first result on the impact of ANG treatment is not particularly surprising. It also warrants a more in-depth scrutiny on the bases of individual tRNA sequences and modification sites.

### 3.4 tRF hypomethylation is not global, but site-specific

**Fig. 7:**
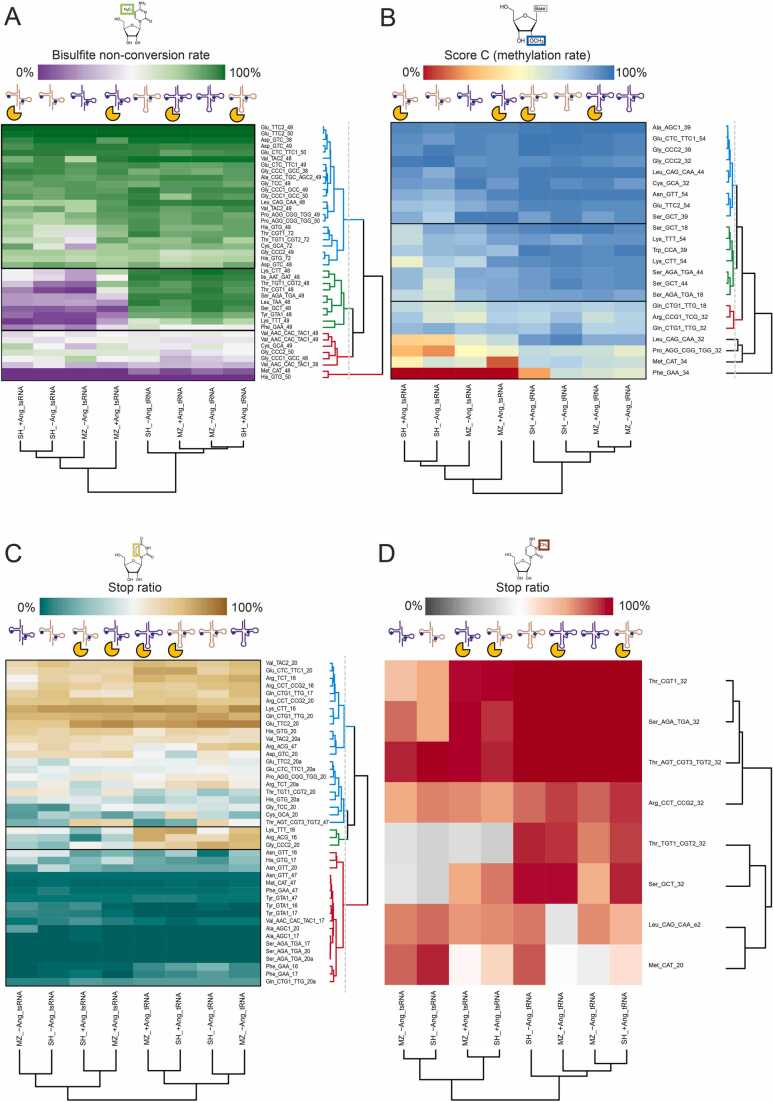
Heatmaps for the assessment of modification changes in individual modified sites in tRNAs/tsRNAs. Each heatmap displays one specific modification’s stoichiometry across the different samples (in X-axis) and the different sites retained for analysis (in Y-axis). Panel A displays m5C stoichiometry through bisulfite non-conversion rate (NonConv) from BisulfiteSeq, Panel B is for 2’-O-methylation through MethScore (also known as Score C) from RiboMethSeq and Panels C, D, and E are respectively for m7G, m3C and D though stop ratio from AlkAnilineSeq (see Material and Methods for further details). This stoichiometry is color-coded depending on the detection method used. Each sample and each site are hierarchically clustered based on the Euclidean distances between each subset, represented by dendrograms at the bottom for samples and on the right for sites. Dendrograms have been colored based on 3 main cluster properties: blue for sites with high stoichiometry and low variation, red for sites with low stoichiometry and low variation and green for sites with high stoichiometry variability. 4 sites remained unclassified for RiboMethSeq due to their unique behavior.

Following up on the global conclusions drawn from the data representation in Fig. 5, namely significant differences between tRNAs and tsRNAs, and moderate impact of ANG treatment, data were scrutinized at the level of individual tRNA species. The corresponding heatmaps are displayed in Fig. 7. Interestingly, clustering of the m^5^C sites (Fig. 7A) readily yields three categories that characteristically differ with regard to modification status and its variation. These comprise (i) sites with high methylation stoichiometry and low variation, (ii) low methylation levels with low variation, and (iii) average methylation levels with substantial variation within the different cell lines, RNA species, and angiogenin treatment status. Category (i) has stable modification levels even after processing or degradation into tsRNAs, suggesting little effect of the modification on the processing event. Similarly, a lack of variation in stoichiometry as in category (ii) would also be taken as a lack of impact of the modification on tRF generation. Therefore, of highest interest among these are data from category (iii), because they comprise data from a dynamic range that responds to either native processing of tRNAs into tsRNAs or to exogenous angiogenin.

Accordingly, in Fig. 7A, the heatmap for m^5^C levels shows category (i) species on the top on mostly dark green (high methylation) and category (ii) on the bottom, featuring mostly light green/purple (low methylation). Sandwiched between these is category (iii) in the middle, its dynamic stoichiometry visualized by the presence of distinct shades of green and purple in each row. For m^5^C, the 10 members of category (iii) include tRNA isoacceptors for Ser (2 species), Lys (2), Thr (2) and one each for Leu, Ile, Phe and Tyr. Interestingly, the sites concerned are exclusively at positions 48 and 49, and these sites are typical substrates of NSUN2. A tentative interpretation, to be discussed later in more detail, posits that these tRNAs are moderately good substrates of NSUN2, i.e. better substrates than are tRNAs from category (ii), but weaker substrates than tRNAs from category (i), and that this property intervenes in regulation of their stability.

A similar division of Nm sites is illustrated in a section of the heatmap in Fig. 7B, where category (iii) comprises a total of 7 sites in tRNAs which are coding for Ser (4 sites), Lys (2) and one for Trp. In contrast to m^5^C, the distribution of sites is heterogeneous throughout the tRNA, including sites at positions 18, 39, 44 and 54, thus all sites depicted in Fig. 3 except for those in the anticodon loop.

A division into three categories for the mapping from AlkAnilineSeq turned out to be meaningful only for D residues. The heatmap for D residues is shown in Fig. 7 C. Here, we have identified 3 tRNA sequences that show high modification stoichiometry in tRNAs and a clear drop in tsRNAs, namely isoacceptors coding for Lys, Arg, and Gly. The results for the only 8 m^3^C sites are shown in Fig. 7D. Given the low number, categorization was deemed not meaningful and sequences were assessed on an individual basis instead. Here a Thr isoacceptor showed a clear decrease of m^3^C stoichiometry between tRNAs and tsRNAs, and a Ser isoacceptor showed high modification in tRNAs, and a response to ANG in the tsRNAs. Furthermore, essentially all m^7^G residues were completely modified, rendering a comparative analysis useless in the objective of this work. The corresponding heatmap for m^7^G is therefore shown in the supplement (Fig. S7).

Altogether the above analyses identified 20 variable sites in category (iii), as listed in Table 1. Interestingly, in all these cases, the tRNAs were more highly modified than were the tsRNAs. This has induced us to re-examine the heatmaps for cases, in which the tsRNAs were more highly modified than the tRNAs. One could indeed make two weak cases along this line (namely m^5^C and m^3^C), but the tsRNAs modification stoichiometry is never consistently high. Indeed, the only cases where tsRNAs reach maximum modification stoichiometry are such, where the same is true for the corresponding full length tRNAs.

**Table 1:** Category (iii) sites show high absolute values for correlation factors for PC1.

	PC1 loadings	PC2 loadings	PC3 loadings	PC4 loadings	Modification
Thr_CGT1_48	-0.99	-0.09	0.07	0.02	m^5^C
Ser_AGA_TGA_49	-0.98	0.10	0.09	-0.02	m^5^C
Lys_TTT_49	-0.98	0.14	0.05	0.06	m^5^C
Tyr_GTA1_48	-0.97	0.09	0.12	0.04	m^5^C
Lys_CTT_48	-0.97	-0.12	0.16	0.04	m^5^C
Ile_AAT_GAT_48	-0.96	0.03	0.27	0.05	m^5^C
Thr_TGT1_CGT2_48	-0.95	-0.23	0.07	0.00	m^5^C
Leu_TAA_48	-0.94	0.17	0.11	-0.03	m^5^C
Ser_GCT_49	-0.94	0.18	0.09	0.02	m^5^C
Phe_GAA_49	-0.94	-0.08	0.11	-0.28	m^5^C
Trp_CCA_39	-0.91	0.29	0.02	-0.13	Nm
Arg_ACG_16	-0.90	0.38	-0.09	0.00	D
Gly_CCC2_20	-0.89	-0.27	0.14	0.06	D
Lys_CTT_54	-0.86	0.15	-0.08	-0.31	Nm
Ser_AGA_TGA_18	-0.85	-0.15	-0.49	-0.05	Nm
Ser_AGA_TGA_44	-0.84	-0.24	-0.41	0.06	Nm
Ser_GCT_44	-0.81	-0.35	-0.42	0.09	Nm
Lys_TTT_54	-0.80	0.39	-0.14	-0.19	Nm
Ser_GCT_18	-0.79	0.32	-0.14	-0.38	Nm
Lys_TTT_16	-0.75	0.57	-0.17	0.12	D

**Fig. 8:**
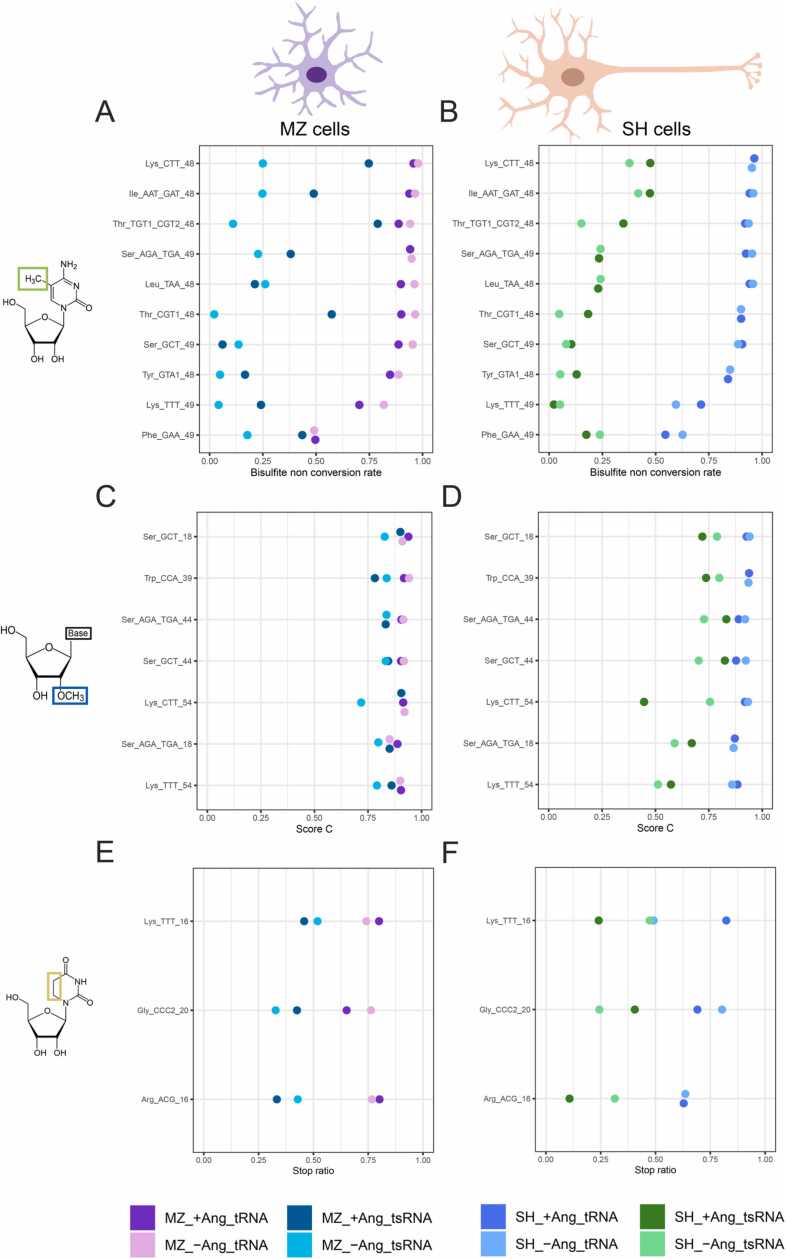
Dot plots of the various sites labeled as category (iii) from the heatmaps. Left panels (A, C, E) for MZ cells, right panels (B, D, F) for SH cells. The nature of modified nucleotide is shown on the left, and the identity of tRNA/tsRNA site on the left of each panel. Color code indicates the identity of the sample (tRNA/tsRNA) and treatment with ANG.

Among the above cited 20 sites that are in category (iii), Isoacceptors decoding Ser (6 sites) and Lys (5) make up more than 50% of the cases. Upon inclusion of Thr (2) and Gly (1) over ⅔ of the cases are covered. Values for category (iii) are given in Fig. 8, which also allows a visual verification of the claims above.

### 3.5 Identification of variable sites by principal component analysis

**Fig. 9:**
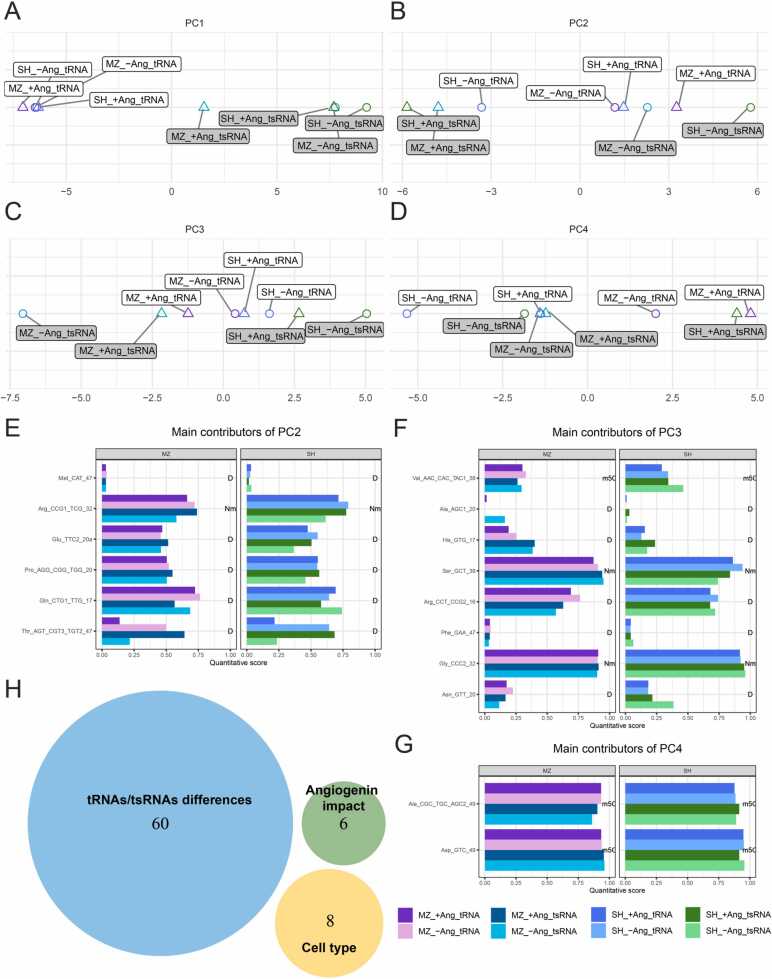
(A-D) Positions of datasets in 4 orthogonal vectors resulting from a PCA of all data points. Each principal component consists of linear combinations of modification data. The numbering PC1 through PC4 is according to the share of data that accounts for data variability: PC1 48.5%, PC2 15.4%, PC3 11.7% PC4 10.7%. (**E-G**) Barplots of the main contributors (eigenvectors) strongly correlating (absolute correlation coefficient > 0.70) with principal components 2, 3 and 4, respectively. (**H**) Venn diagram of the different contributors of the principal components and their interpretation. All PC1 main contributors have been labeled as “tRNAs/tsRNAs differences”, PC2 as “Angiogenin impact” and PC3 as “Cell type”. Main contributors to PC4 show negligible data variability and their information content is therefore negligible for the case at hand.

Following our rationale, that differences are to be found at sites of maximum variability, we set out to identify these using a principal component analysis (PCA). Fig. 9 shows the positions of 8 datasets (MZ vs. SH, tRNA vs tsRNA, -ANG vs. ANG+) along four orthogonal vectors resulting from a PCA from all datapoints used in Fig. 7, i.e. including all modification mapping methods. The PC1 vector, representing the majority of variability in the data (48.5%, Fig. 9A) allows a very clear distinction between tRNAs (negative coordinate to the left) and tsRNAs (to the right), thereby confirming our previous conclusions from statistical analysis (Fig. 5 & 7). We extracted the principal contributors to PC1 according to their correlation/anticorrelation coefficients with the 4 PCs (see table S7). Table 1 lists category (iii) sites, as obtained from clustering in heatmaps, along with their correlation coefficients. Clearly visible, all category (iii) sites also feature high absolute values for correlation factors for PC1. A number of these sites, especially Thr CGT1 m^5^C48, as highlighted in Table 1, feature low correlation with the other 3 PCs, which are orthogonal to PC1, identifying them as potential markers for the distinction of tsRNAs from tRNAs. Taken together, this effectively validates our approach, to identify variable sites of biological significance by PC vector analysis.

From further inspection of the four PC vectors in Fig. 9, PC3 separates the two cell lines under investigation: MZ to the left and SH to the right (Fig. 9C), with the separation of tsRNAs being more pronounced. Principal contributors of PC3, as listed in Table S7 mark differences in tRNA modification stoichiometry characteristic between these cell lines. PC2 emerges as the vector harboring some information on the effect of ANG treatment on tsRNAs (Fig. 9B), which are well separated in the PC2 dimension, but this does not apply to tRNAs. PC4 again shows a uniform response to ANG treatment for each sample, in that the treatment leads to a positive shift along the PC4 vector for each tRNA MZ, tRNA SH, tRF MZ and tRF SH. Indeed, a combination of PC2 and PC4 is suited to somewhat separate samples according to their exposure to ANG (Fig. S8), although there is no strong clustering clearly visible. The latter indicates that the response to ANG treatment is not very pronounced, and not characteristic.

Barplots with of modification stoichiometries of the main contributors (eigenvectors) strongly correlating (absolute correlation coefficient > 0.70) with principal components 2, 3 and 4, are shown in Fig. 9 E,F, and G. Given that PC1 features 60 such contributors, the corresponding barplots were moved to the supplement (Fig. S9). Also, category (iii) sites are a subset of the latter, and some values are provided in Fig. 8. Using a correlation cutoff of 0.7 leads to a clean separation, meaning that no strong contributors to PC1 feature among the strong contributors to the other PCs, and vice versa. This orthogonality is visualized in the Venn diagram in Fig. 9H, showing no overlap between what we herewith identify as marker sets/signatures for the three biological input parameters: RNA type (tRNA/tsRNA), exposure to angiogenin (-ANG/+ANG) and biological origin (MZ/SH cells).

## 4 Discussion

### 4.1 Towards a complete map of modifications in human tRNAs

There are several reasons why the topic of substoichiometric modification has come under more intensive investigation in recent years. Foremost among these, is probably the understanding that modification levels in tRNAs and other RNAs, including mRNAs, are subject to dynamic changes, associated with a new level of regulation of gene expression. The view of continuous, stimulus-dependent fine-tuning of RNA properties on the level of individual molecules replaced the picture of modification as an obligate maturation step to be invariantly undergone by every single RNA molecule of a given RNA species. Another strong contribution came from detection and sequencing technology, which enabled quantification of more than single modification sites, and which made investigations with large data sets feasible. While applications of mass spectrometry in RNA modification research have also surged, this applies even more to modification mapping techniques based on high throughput sequencing. Given the plethora of such mapping methods, we had to limit the number applied here for practical as well as financial reasons. The joint use of BS-Seq, RiboMethSeq and AlkAnilineSeq here was governed by the following rationale. Firstly, m^5^C and Nm residues both occur abundantly and at numerous positions in tRNA, and moreover have both been reported to affect tRNA cleavage into tsRNAs [53], [54], hence it was decided to cover both of these by methods well established in our labs. Secondly, AlkAnilineSeq offered an attractive combination of features, such as low requirement for RNA material (∼50 ng per sample), very low background, and the simultaneous mapping of m^3^C, m^7^G and D residues. The latter in particular are known to occur sub-stoichiometrically and are therefore prime candidates for yielding information to address our key question. Indeed, our data show a majority of D-sites to be substoichiometric as well as dynamic, and a number of them also featured among the top contributors of PC vectors.

Despite decades of tRNA studies, the complete human tRNA dataset was never entirely explored and only the global location of modified sites was firmly established (tRNAdb [45] /Modomics [55]/gtRNAdb [56]). Comprehensive analysis by the Suzuki lab established the full profile of human mitochondrial tRNAs modifications [57], [58], but almost ½ of cytoplasmic tRNA species have never been systematically studied.

### 4.2 Limitations of the current study and its methodology

Our combined mapping efforts identified a total of 149 sites in human tRNAs, with more than half of these not previously reported. This is not surprising, since less than half of human cytosolic tRNAs have been exhaustively analyzed with respect to their modification content. Literature on similarly conservative approaches reports False Discovery Rates (FDR) in the single digit percentile and False Negative Rates (FNR) that are much higher. FDR examples are about 7% for Ribomethseq (RMS, [29], [59]) with FNR about 40%, FDR for Alkaniline seq better than 4% ([30], FNR ∼53%), and FDR for bisulfite seq also about 7% ([60], FNR∼4%). This data is in keeping with our unpublished datasets e.g. on mammalian rRNA. In comparison, when regarding the flowchart in Fig. S4, we find that plausibility considerations applied to the present dataset suggest FDR rates of 13% (RMS), 4% (AAS) and 8% (BS), while FNRs are again higher at 54%, 53% and 10%, respectively. This data argues for 149 being a number that is likely still incomplete due to our deliberately high thresholds. Yet at present it is still an expansion of the previous census, and the most complete compilation of m^5^C, Nm, m^3^C, m^7^G and D residues in this tRNA population.

### 4.3 Information content and biological idiosyncrasy of tRNA modification patterns

The principle research question driving our investigations was whether tRNA modification patterns contain information reflecting the physiological state of a cell, to a degree that different cell types could be distinguished from one another. Given that even differentiated cells have essentially the same set of tRNA sequences and genes for modification enzymes, such differences would likely be reflected, not in the presence or absence of a specific (marker) modification, but in the degree to which a certain position within the tRNA was modified. Information on the identity of a cell would therefore have to be extracted from the entirety of substoichiometric modifications present in tRNAs and their processing products, i.e. tRNA fragments of a cell. Inspired by the finding that tRNA modification patterns could be discriminated in different mouse brain tissues [23], we reasoned that this might be possible for different neuronal cell lines as well. We do indeed see distinct modification signatures that allow a separation of the two cell lines investigated in a PCA analysis. Interestingly, that separation was less pronounced than the difference between tRNAs and tsRNAs. To gauge the degree to which our data allowed to distinguish the influence of the principle parameters RNA species (tRNA vs. tRF, cell type, ANG treatment), we conducted a PCA. Roughly 50% of data variability was represented in PC1, whose faithful separation of tRNAs from tsRNAs could be verified by inspection of clustering in heatmap data, and visual inspection of the most variable sites, i.e. those belonging to category (iii). Distinction of ANG treatment resides in PC2 (vide infra) and of cell types in PC3, both containing a much smaller fraction of data variability. Finally, PC4 did not provide much additional distinction, and a look at its principal contributor in Fig. 9 G reveals that their modification stoichiometry barely shows variation. From this we conclude that parameters contained in PC4 are too close to background noise to be meaningfully interpreted. We will thus focus on PC1, PC2 and PC3 here.

### 4.4 tRNA fragments are hypomodified relative to tRNAs

tRNA fragments are generated from full-size mature tRNA molecules by cleavage in the loop regions, the most frequently observed are 5’-tiRNAs and 3’-tiRNAs, representing tRNA halves, but other even shorter tRNA derived species have been reported (referred to as tRFs) [9], [61], [62]. These processing pathways are mediated by different endonucleases with broad specificity. ANG cleavage is mainly discussed here, but other concomitant pathways have been described. For instance, Dicer cleaves tRNAs into miR-like fragments (tRFs) [63], [64] and another Dicer- and ANG-independent pathways have been reported [2], [7], [65]. These multiple and overlapping tRNA cleavage pathways create an impressive number of tRNA-derived small RNAs, thus appealing for more detailed characterization.

Moreover, a number of modifications enzymes and their corresponding modifications have been reported to affect cleavage of tRNAs into tsRNAs. We [5], [6], [66] and others [54] had previously contributed to work on the effect of m^5^C modifications on tRNA processing into tsRNAs by ANG. Comparable protective effects were also reported of Nm modifications in the anticodon loop [53]. Similarly, m^1^A modification and de-modification [67] were reportedly involved.

A question to which this study contributed significantly is how strongly modification profiles of mature tRNAs and their tsRNA processing products differ. The clearest statement to be drawn from all our statistical analyses is that there is a very substantial difference in the stoichiometry of modification between tRNAs and tsRNAs. Visual inspection of category (iii) sites in Fig. 8 consistently finds tsRNAs hypomodified relative to the corresponding tRNAs. This effect is therefore dominantly reflected in PC1, and accounts for most of the variability in the dataset, which encompasses the vast majority of category (iii). The correlation of differences between tRNA and tsRNAs to PC1 makes this effect the strongest of the observed ones, while smaller shares of data variability account for PC2, PC3 and PC4.

An important aspect of data interpretation is orthogonality between the four PCs, and the correlation coefficients of a modification site’s Eigenvector with any of them. An instructive example is found at the top of Table 1: The m^5^C48 site in tRNAThr_CGT1 shows near perfect anti-correlation with PC1 (−0.99), and low correlation to all its orthogonal vectors. Its near complete alignment with the PC1 vector means that changes of m^5^C48 occur principally between tRNAs and tsRNAs. This interpretation is readily underscored by Fig. 8, which shows that tRNAs consistently show high amounts of m^5^C48, whereas tsRNAs are consistently low. This confers to this modification a diagnostic value for the discrimination of tRNAs vs. tsRNAs.

Of note, the measured levels of modifications and tsRNAs are observed in living cell, where they are part of a flow that is not only governed by tsRNA generation through tRNA cleavage, but also by depletion of the tsRNA pool during further processing or degradation, making predictions or conclusions as to the effect of modifications difficult. Thus, our finding of tsRNAs being hypomodified relative to the mature tRNAs is not trivial or even easily anticipated, since the tsRNA likely have a short lifetime, possibly shorter than even hypomodified tRNAs.

### 4.5 Processing of modified tRNA into fragments by ANG

In the context of neuronal cells and tRNAs, their processing by ANG into tsRNAs is a matter of high interest given the involvement of ANG in various neuronal diseases [9], [68]. Most studies that originally identified connections between tRNA modification and ANG cleavage were conducted in an all-or-nothing setting, i.e. in knockout model organisms, where more subtle effects of ANG would go unnoticed [5], [6], [66]. Other studies aimed at defining the cleavage preferences of ANG in complex tRNA mixtures were performed in vitro [9], [69], therefore not accounting for the steady state arising from *de novo* tRNA synthesis, its modification, and its degradation and processing into tsRNAs. In the present setting, we saw the opportunity to implement exactly this, by applying exogenous ANG *in cellulo*, followed by analysis of tRNA modification stoichiometry.

Visual inspection revealed many of our 149 modification sites as dynamically affected by ANG treatment. For example, data in Fig. 8A confirm that m^5^C levels vary upon ANG treatment in a number of sites in category (iii), and the same applies to Nm in Fig. 8B and D residues in Fig. 8C. However, since many of these sites strongly correlate with PC1 and thus the tRNAs/tsRNAs differences, meaning that their modification stoichiometry is defined not only by ANG but also by other parameters, meaning that these sites hold limited information of diagnostic value.

Interestingly, a perusal of main contributors of PC2 implies that several sites of D modification are significantly affected by ANG treatment. These sites show limited to no difference between tRNA and tsRNAs, which is in line with PC2 being orthogonal to PC1. While variation of most sites is moderate, D47 in tRNA^Thr^(HGT) shows a consistent response to ANG treatment, in that tRNA modification level decreases while tsRNAs show concomitant increase of D level in both cell lines. Accordingly, these characteristics are independent of the tRNAs/tsRNAs discrimination of PC1, and hold diagnostic value for identification of ANG treatment. Dihydrouridine has so far not been implied in tRNA cleavage, presumably because methods for high throughput mapping of D have only recently become available [70], [71].

While the case of D47 is to be verified by further experiments, it features highly interesting aspects of ANG mediated cleavage as follows. Pending experimental verification by orthogonal measurement, this might be a case of stimulation of ANG-mediated cleavage by modifications in the structural core of the tRNA, where other modifications have been shown to have protective effects against cleavage. This is plausible with regard to the known biophysical and biochemical effects of modifications on tRNA structure: m^5^C and Nm are known to stabilize tRNA structure overall, as well as locally against cleavage. In contrast, D is thought to render tRNA structure more flexible, making it more prone to cleavage [14], [15]. Further study, beyond the scope of this paper, is required to tease out the mechanistic aspects of this intriguing observation.

## CRediT authorship contribution statement

**Florian Pichot:** Conceptualization, Data curation, Formal analysis, Software, Validation, Visualization, Project administration. **Marion C. Hogg:** Conceptualization, Data curation, Formal analysis. **Virginie Marchand:** Conceptualization, Data curation, Formal analysis. **Valérie Bourguignon:** Conceptualization, Data curation, Formal analysis. **Elisabeth Jirström:** Conceptualization, Data curation, Formal analysis. **Cliona Farrell:** Conceptualization, Data curation, Formal analysis. **Hesham A. Gibriel:** Conceptualization, Data curation, Formal analysis. **Jochen H. M. Prehn:** Conceptualization, Funding acquisition, Investigation, Methodology, Supervision, Writing – original draft, Writing – review & editing. **Yuri Motorin:** Conceptualization, Funding acquisition, Investigation, Methodology, Supervision, Writing – original draft, Writing – review & editing, Project administration. **Mark Helm:** Conceptualization, Funding acquisition, Investigation, Methodology, Supervision, Writing – original draft, Writing – review & editing, Data curation, Formal analysis. All authors have read the manuscript and agree with its content.

## Conflict of interest

Mark Helm is a consultant for Moderna Inc.
