# Supplementary material for: Quantification of substoichiometric modification reveals global tsRNA hypomodification, preferences for angiogenin-mediated tRNA cleavage, and idiosyncratic epitranscriptomes of human neuronal cell-lines
Source: Comput Struct Biotechnol J. 2022 Dec 19;21:401–17. doi: 10.1016/j.csbj.2022.12.020 (PMC9798144; doi:10.1016/j.csbj.2022.12.020)
Supplement: Supplementary file 1 — Supplementary material [file mmc1.pdf]

Supplemental Material to:

Quantification of substoichiometric tRNA modification sites reveals global hypomodification of tsRNA, indicates preferences for angiogenin-mediated tRNA cleavage, and points to idiosyncratic tRNA epitranscriptomes of human neuronal cell lines

Florian Pichot[1,2], Marion C. Hogg[3,#], Virginie Marchand[2], Valérie Bourguignon[2,4], Elisabeth Jirström[3], Cliona Farrell[3], Hesham A. Gibriel[3], Jochen H. M. Prehn[3], Yuri Motorin[2,4,*], and Mark Helm[1,*]

[1] Institute of Pharmaceutical and Biomedical Sciences, Johannes Gutenberg-University Mainz, Staudingerweg 5, 55128 Mainz, Germany.

[2] Université de Lorraine, CNRS, INSERM, IBSLor (UAR2008/US40), Epitranscriptomics and RNA Sequencing Core Facility, F54000 Nancy, France.

[3] Department of Physiology and Medical Physics and SFI FutureNeuro Research Centre, Royal College of Surgeons in Ireland, St. Stephen's Green, Dublin, D02 YN77, Ireland

[4] Université de Lorraine, CNRS, IMoPA (UMR7365), F54000 Nancy, France.

[#] Present address: School of Science and Technology, Nottingham Trent University, Nottingham, NG1 4FQ, United Kingdom

* Correspondence to mhelm@uni-mainz.de or yuri.motorin@univ-lorraine.fr

Table S1. List of 149 tRNA modification sites for assessment of stimulus-dependent modification dynamics. A list of all discarded candidates is available in a separated file called "Supp_Table_all_discarded_candidates.csv"

**tRNA isoacceptor**	**Absolute position from 5'-end**	**Relative position according to tRNA structure**	**Modification**	**Sites attribution**
Ala_AGC1	17	17	D	False negative
Ala_AGC1	20	20	D	False negative
Ala_AGC1	32	32	Nm	False negative (tRNAdb2009 + TARBP1/FTSJ3)
Ala_AGC1	39	39	Nm	True positive (tRNAdb2009)
Ala_AGC1	46	46	m7G	True positive
Ala_CGC_TGC_AGC2	45	46	m7G	Putative site
Ala_CGC_TGC_AGC2	48	49	m5C	Putative site
Arg_CCG1_TCG	32	32	Nm	False negative (TARBP1/FTSJ3)
Arg_ACG	16	16	D	Putative site
Arg_ACG	32	32	Nm	False negative (TARBP1/FTSJ3)
Arg_ACG	47	47	D	Putative site
Arg_CCT_CCG2	16	16	D	Putative site
Arg_CCT_CCG2	20	20	D	Putative site

Arg_CCT_CCG 2	32	32	m3C	True positive
Arg_TCT	16	16	D	Putative site
Arg_TCT	20	20a	D	Putative site
Arg_TCT	46	46	m7G	Putative site
Asn_GTT	16	16	D	False negative
Asn_GTT	20	20	D	False negative
Asn_GTT	47	46	m7G	True positive
Asn_GTT	48	47	D	False negative
Asn_GTT	55	54	Nm	Putative site
Asp_GTC	19	20	D	Putative site
Asp_GTC	38	38	m5C	Putative site
Asp_GTC	47	48	m5C	Putative site
Asp_GTC	48	49	m5C	Putative site
Cys_GCA	19	20	D	Putative site
Cys_GCA	31	32	Nm	False negative (TARBP1/FTSJ3)
Cys_GCA	45	46	m7G	Putative site
Cys_GCA	48	49	m5C	Putative site
Cys_GCA	71	72	m5C	Putative site
Gln_CTG2	48	49	m5C	Putative site

Gln_CTG1_TTG	16	17	D	Putative site
Gln_CTG1_TTG	17	18	Nm	False negative (tRNAdb2009 + TARBP1/FTSJ3)
Gln_CTG1_TTG	19	20	D	True positive
Gln_CTG1_TTG	20	20a	D	False negative
Gln_CTG1_TTG	32	32	Nm	False negative (tRNAdb2009 + TARBP1/FTSJ3)
Gln_CTG1_TTG	48	49	m5C	True positive
Gln_CTG1_TTG	49	50	m5C	True positive
Glu_CTC_TTC1	19	20	D	True positive
Glu_CTC_TTC1	20	20a	D	True positive
Glu_CTC_TTC1	48	49	m5C	True positive
Glu_CTC_TTC1	49	50	m5C	True positive
Glu_CTC_TTC1	53	54	Nm	True positive (tRNAdb2009)
Glu_TTC2	19	20	D	Putative site
Glu_TTC2	20	20a	D	Putative site
Glu_TTC2	48	49	m5C	Putative site
Glu_TTC2	49	50	m5C	Putative site

Glu_TTC2	53	54	Nm	Putative site
Gly_TCC	19	20	D	True positive
Gly_TCC	48	49	m5C	Putative site
Gly_CCC2	19	20	D	True positive
Gly_CCC2	31	32	Nm	True positive (TARBP1/FTSJ3)
Gly_CCC2	38	39	Nm	True positive (tRNAdb2009)
Gly_CCC2	47	49	m5C	True positive
Gly_CCC2	48	50	m5C	True positive
Gly_CCC1_GCC	37	38	m5C	True positive
Gly_CCC1_GCC	46	48	m5C	True positive
Gly_CCC1_GCC	47	49	m5C	True positive
Gly_CCC1_GCC	48	50	m5C	True positive
His_GTG	16	17	D	False negative
His_GTG	19	20	D	True positive
His_GTG	20	20a	D	True positive
His_GTG	48	49	m5C	True positive
His_GTG	49	50	m5C	False negative
His_GTG	71	72	m5C	Putative site

Ile_AAT_GAT	47	46	m7G	Putative site
Ile_AAT_GAT	49	48	m5C	Putative site
Ile_TAT	47	46	m7G	Putative site
Leu_TAA	20	20	D	False negative
Leu_TAA	34	34	Nm	False negative (TARBP1/FTSJ3)
Leu_TAA	58	48	m5C	True positive
Leu_CAG_CAA	33	32	Nm	True positive (TARBP1/FTSJ3)
Leu_CAG_CAA	45	44	Nm	True positive (tRNAdb2009)
Leu_CAG_CAA	51	e2	m3C	True positive
Leu_CAG_CAA	58	48	m5C	Putative site
Lys_CTT	16	16	D	Putative site
Lys_CTT	46	46	m7G	Putative site
Lys_CTT	48	48	m5C	Putative site
Lys_CTT	54	54	Nm	Putative site
Lys_TTT	16	16	D	Putative site
Lys_TTT	46	46	m7G	Putative site
Lys_TTT	49	49	m5C	Putative site
Lys_TTT	54	54	Nm	Putative site
Met_CAT	20	20	m3C	True positive

Met_CAT	34	34	Nm	False negative (tRNAdb2009 + TARBP1/FTSJ3)
Met_CAT	46	46	m7G	True positive
Met_CAT	47	47	D	False negative
Met_CAT	48	48	m5C	False negative
Phe_GAA	16	16	D	False negative
Phe_GAA	17	17	D	False negative
Phe_GAA	32	32	Nm	False negative (tRNAdb2009 + TARBP1/FTSJ3)
Phe_GAA	34	34	Nm	False negative (tRNAdb2009 + TARBP1/FTSJ3)
Phe_GAA	46	46	m7G	True positive
Phe_GAA	47	47	D	False negative
Phe_GAA	49	49	m5C	True positive
Pro_AGG_CGG_TGG	19	20	D	Putative site
Pro_AGG_CGG_TGG	31	32	Nm	False negative (TARBP1/FTSJ3)
Pro_AGG_CGG_TGG	45	46	m7G	Putative site
Pro_AGG_CGG_TGG	48	49	m5C	Putative site
Pro_AGG_CGG_TGG	49	50	m5C	Putative site

Ser_AGA_TGA	16	17	D	False negative
Ser_AGA_TGA	17	18	Nm	False negative (tRNAdb2009 + TARBP1/FTSJ3)
Ser_AGA_TGA	19	20	D	False negative
Ser_AGA_TGA	20	20a	D	False negative
Ser_AGA_TGA	32	32	m3C	True positive
Ser_AGA_TGA	44	44	Nm	True positive (tRNAdb2009)
Ser_AGA_TGA	50	e2	m3C	False negative
Ser_AGA_TGA	57	49	m5C	True positive
Ser_GCT	17	18	Nm	False negative (TARBP1/FTSJ3)
Ser_GCT	32	32	m3C	True positive
Ser_GCT	39	39	Nm	Putative site
Ser_GCT	44	44	Nm	Putative site
Ser_GCT	57	49	m5C	Putative site
Thr_CGT1	31	32	m3C	True positive
Thr_CGT1	47	48	m5C	Putative site
Thr_CGT1	71	72	m5C	Putative site
Thr_TGT1_CGT2	19	20	D	Putative site
Thr_TGT1_CGT2	32	32	m3C	Putative site

Thr_TGT1_CGT2	46	46	m7G	Putative site
Thr_TGT1_CGT2	48	48	m5C	Putative site
Thr_TGT1_CGT2	72	72	m5C	Putative site
Thr_AGT_CGT3_TGT2	33	32	m3C	True positive
Thr_AGT_CGT3_TGT2	48	47	D	Putative site
Thr_AGT_CGT3_TGT2	49	48	m5C	Putative site
Thr_AGT_CGT3_TGT2	50	49	m5C	Putative site
Thr_AGT_CGT3_TGT2	73	72	m5C	Putative site
Trp_CCA	31	32	Nm	False negative (TARBP1/FTSJ3)
Trp_CCA	33	34	Nm	False negative (TARBP1/FTSJ3)
Trp_CCA	38	39	Nm	Putative site
Trp_CCA	45	46	m7G	Putative site
Tyr_GTA1	16	16	D	False negative
Tyr_GTA1	17	17	D	False negative
Tyr_GTA1	46	46	m7G	True positive
Tyr_GTA1	47	47	D	False negative
Tyr_GTA1	48	48	m5C	True positive

Val_AAC_CAC_TAC1	16	17	D	False negative
Val_AAC_CAC_TAC1	19	20	D	False negative
Val_AAC_CAC_TAC1	20	20a	D	False negative
Val_AAC_CAC_TAC1	38	38	m5C	Putative site
Val_AAC_CAC_TAC1	46	46	m7G	True positive
Val_AAC_CAC_TAC1	47	47	D	False negative
Val_AAC_CAC_TAC1	48	48	m5C	Putative site
Val_AAC_CAC_TAC1	49	49	m5C	Putative site
Val_TAC2	19	20	D	Putative site
Val_TAC2	20	20a	D	Putative site
Val_TAC2	46	46	m7G	Putative site
Val_TAC2	48	48	m5C	True positive
Val_TAC2	49	49	m5C	True positive

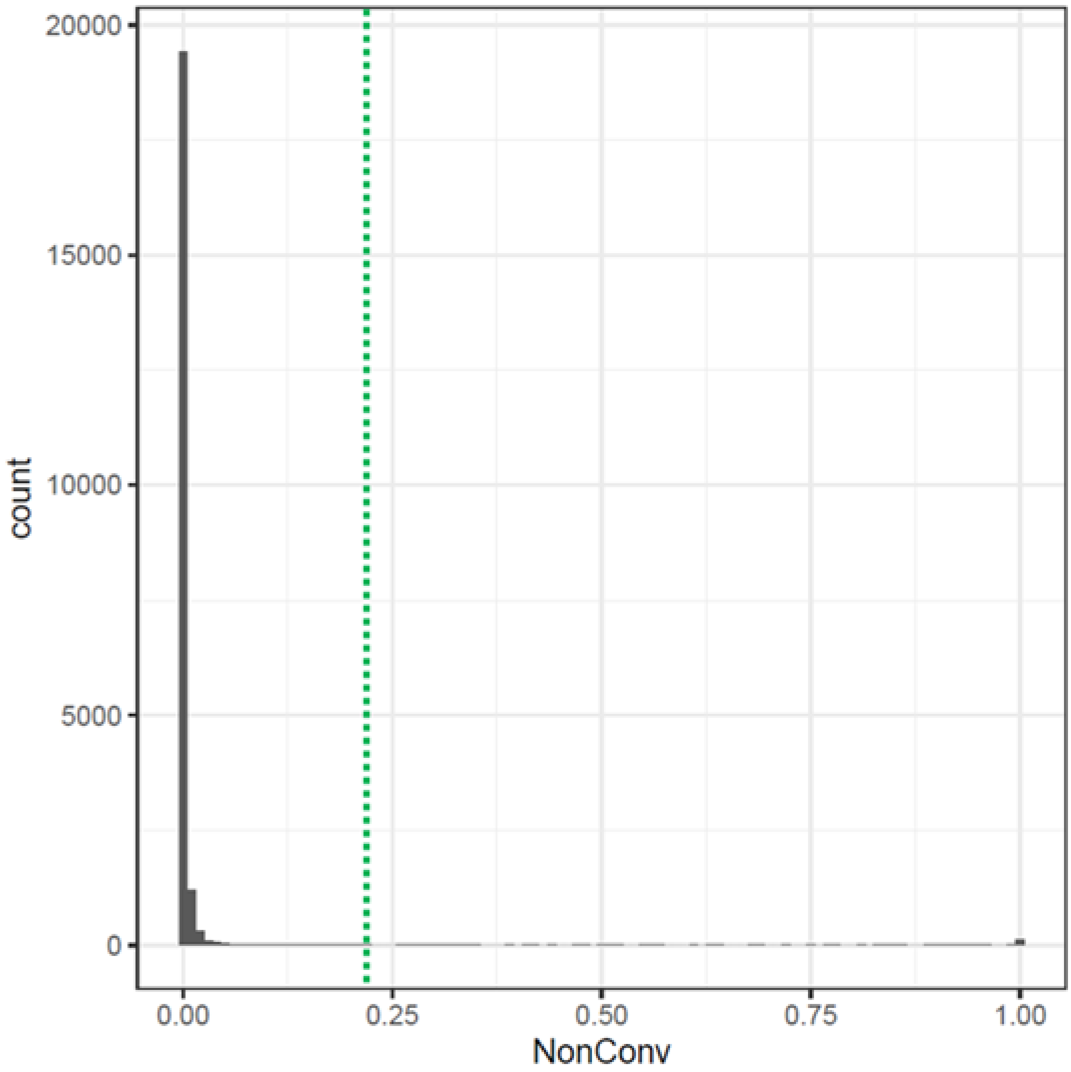

Figure S1 : Noise estimation of Bisulfite Non-conversion rate from Bisulfite sequencing. Each bar represents the counts of non-m5C sites (all rRNA C sites except 2 known positions in rRNA 28S) according to their bisulfite non conversion rate (NonConv). The distribution of these non-m5C sites, considered as noise, helped setting up a detection threshold at 0.228, corresponding to the average plus 3 times the standard deviation of the noise distribution, highlighted here by a green vertical bar.

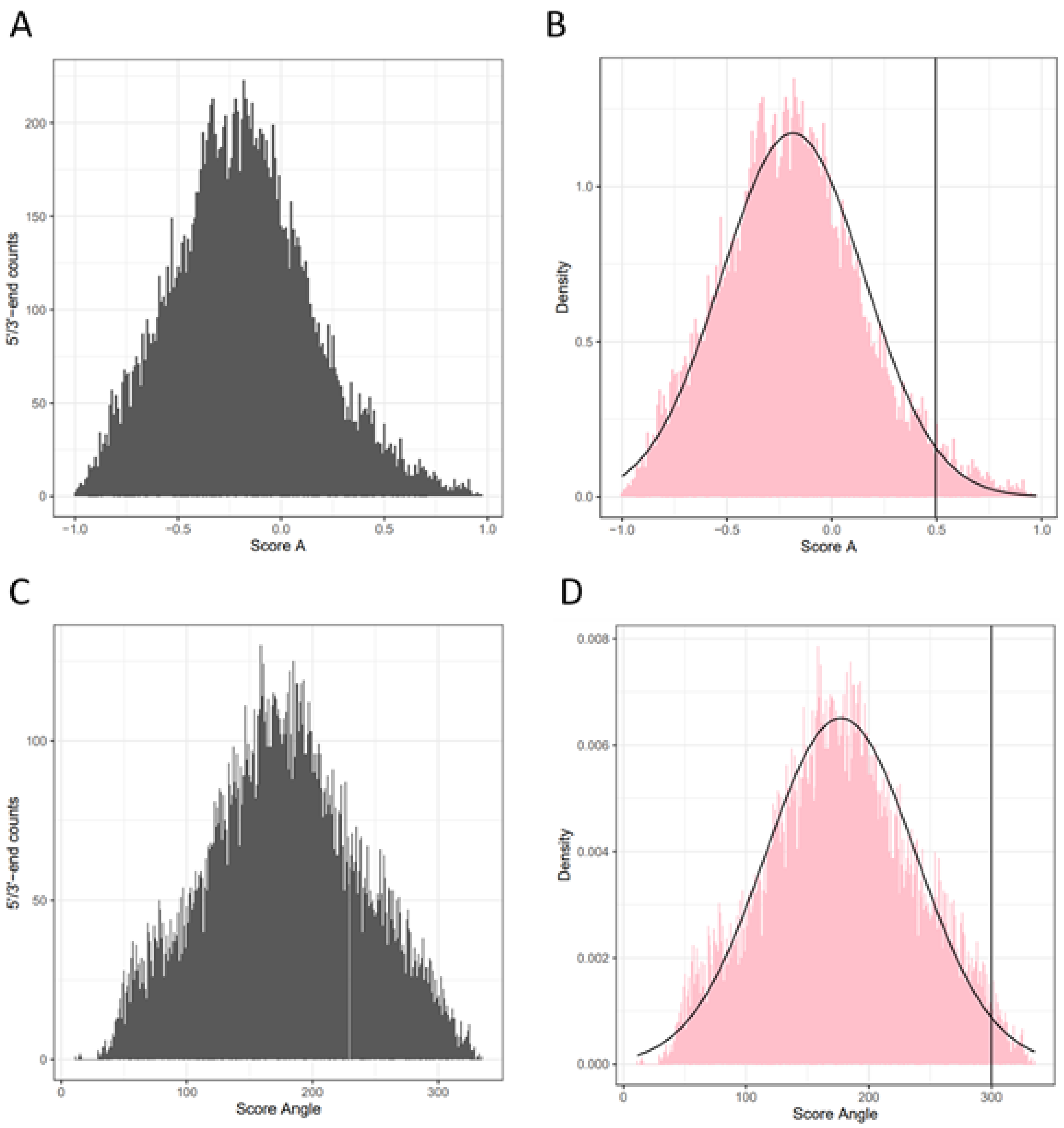

Figure S2 : Noise estimation of the different RiboMethScores from RiboMethSeq. Panels **(A, C)** displays non 2'-O-methylated sites (all tRNA sites not located at position 18, 32, 34, 39, 44 and 54) count distribution according to score A and score Angle, respectively. Panels **(B, D)** display fitting of noise distribution to a Gaussian distribution for score A and score Angle, respectively. The vertical bar indicates the threshold used for both scores which is at the noise average plus 2 times the noise standard deviation, which is 0.495 for score A and 300 for score Angle.

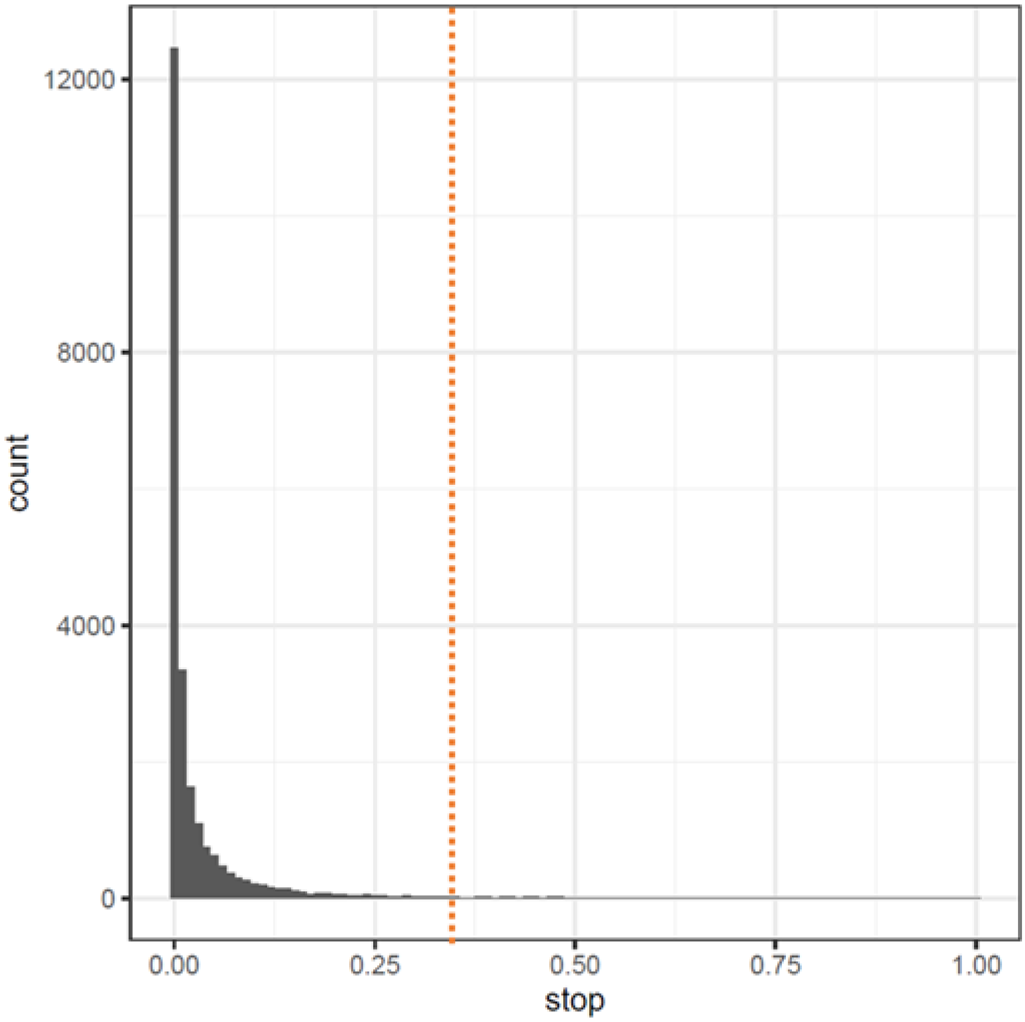

Figure S3 : Noise estimation of stop ratio from AlkAnilineSeq.

Each bar represents the counts of sites unseen by AlkAnilineSeq (no m7G, m3C ou D known enzymatic positions) according to their stop ratio (stop). The distribution of these undetectable sites has been considered as noise for detection threshold characterisation. Threshold determination through noise average + 3* noise standard deviation application occurs within a range from 0.263 (For A sites) to 0.418 (For G sites), with a default threshold of 0.353 while taking in account all nucleotides (represented here by an orange vertical bar).

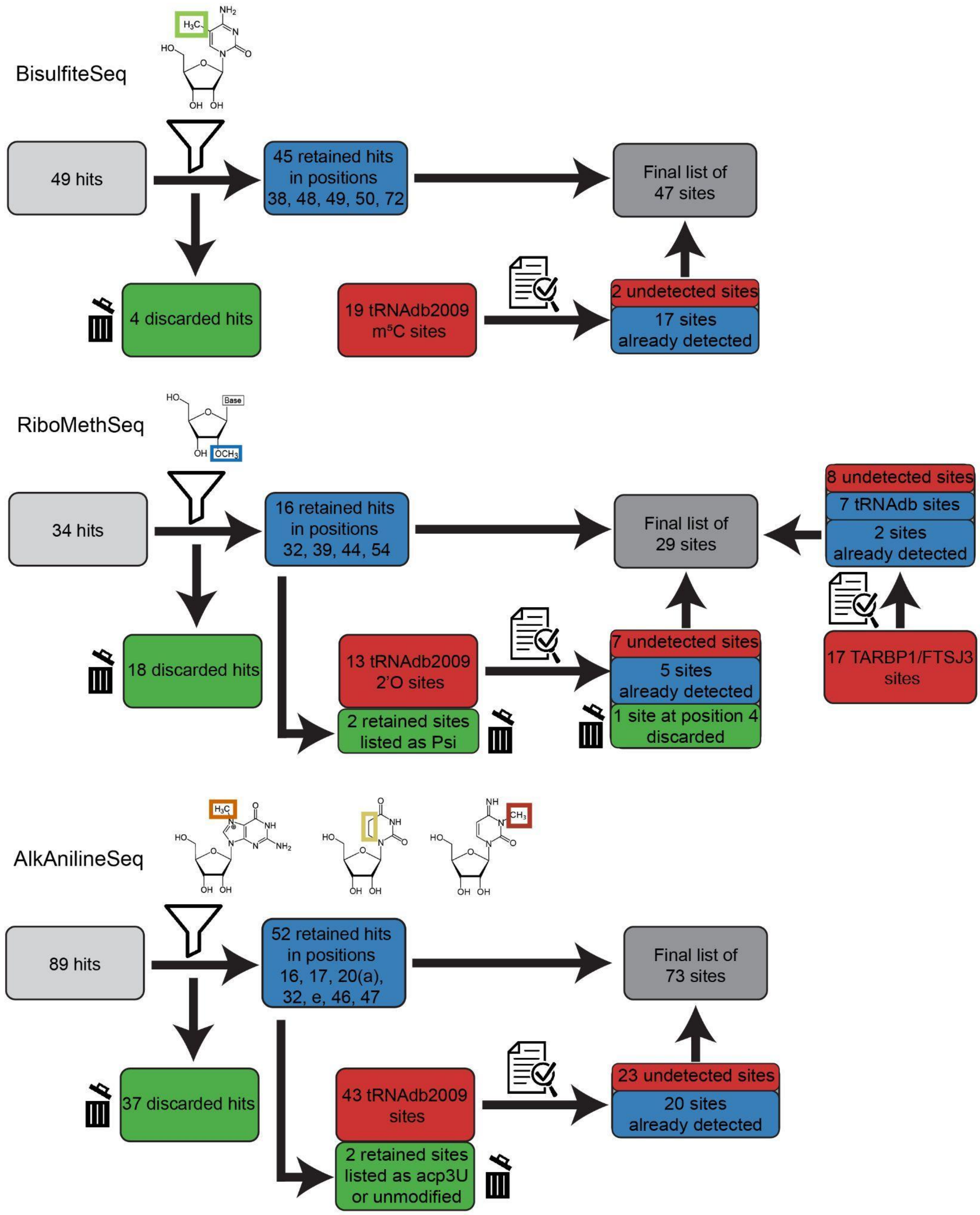

Figure S4 : Flow-chart on sites selection. Counting of retained and discarded hits along the analyses of the three different sequencing methods. On top, the sites selection for Bisulfite sequencing started with 49 total hits where 4 of them didn't belong to known m5C sites. After comparison with tRNAdb2009, 2 sites have been missed and have been added back to the list. For RiboMethSeq in the middle, out of the 34 initial hits, 18 didn't belong to known 2'-O-methylated sites. After comparison with tRNAdb2009 and two papers from the literature (Marchand et al., 2017 and Angelova and al. 2020) with validated sites for Gm18 and Nm32/34, 16 undetected

sites have been added back and 2 hits which are referenced as pseudouridine residues have been discarded. Finally, for AlkAnilineSeq at the bottom, 37 hits have been discarded from the 89 initial ones. Then, by comparing our hits with tRNAdb2009, 23 known sites (mainly dihydrouridine ones) has been missed and added back, while 1 hit which was originally retained has been identified as acp3U and another 1 was known as unmodified. The list of all discarded candidates is available in a separate file called "Supp_Table_all_discarded_candidates.csv"

Tables S2-S6 Wilcoxon's text for assessment of significative differences between datasets

All p-values below have been calculated with the function "wilcox.test" from the *stats* R package with default parameters, which means the alternative hypothesis tested here is "two.sided".

Table S2A : Significant differences estimation for m5C without cell line distinction.

	-Ang_tRNA	-Ang_tRF	+Ang_tRNA	+Ang_tRF
-Ang_tRNA	1			
-Ang_tRF	<0.0001 ****	1		
+Ang_tRNA	0.3966	0.0002 ***	1	
+Ang_tRF	0.0003 ***	0.2058	0.004 **	1

Table S2B : Significant differences estimation for m5C with cell line distinction.

	MZ_-Ang_tRNA	SH_-Ang_tRNA	MZ_-Ang_tRF	SH_-Ang_tRF	MZ_+Ang_tRNA	SH_+Ang_tRNA	MZ_+Ang_tRF	SH_+Ang_tRF
MZ_-Ang_tRNA	1							
SH_-Ang_tRNA	0.9646	1						
MZ_-Ang_tRF	0.0012 **	0.0011 **	1					
SH_-Ang_tRF	0.004 **	0.0065 **	0.7278	1				
MZ_+Ang_tRNA	0.2081	0.2339	0.0154 *	0.0406 *	1			
SH_+Ang_tRNA	0.7534	0.7444	0.0008 ***	0.0023 **	0.1318	1		
MZ_+Ang_tRF	0.0252 *	0.0258 *	0.1431	0.2978	0.2552	0.0175 *	1	
SH_+Ang_tRF	0.0035 **	0.0041 **	0.4420	0.8192	0.0406 *	0.0019 **	0.4091	1

Table S3A : Significant differences estimation for Nm without cell line distinction.

	-Ang_tRNA	-Ang_tRF	+Ang_tRNA	+Ang_tRF
-Ang_tRNA	1			
-Ang_tRF	0.0015 **	1		
+Ang_tRNA	0.6181	0.0039 **	1	
+Ang_tRF	0.003 **	0.6887	0.0129 *	1

Table S3B : Significant differences estimation for Nm with cell line distinction.

	MZ_-Ang_tRNA	SH_-Ang_tRNA	MZ_-Ang_tRF	SH_-Ang_tRF	MZ_+Ang_tRNA	SH_+Ang_tRNA	MZ_+Ang_tRF	SH_+Ang_tRF
MZ_-Ang_tRNA	1							
SH_-Ang_tRNA	0.2043	1						
MZ_-Ang_tRF	0.1823	0.0228 *	1					
SH_-Ang_tRF	0.0294 *	0.0019 **	0.5924	1				
MZ_+Ang_tRNA	0.9814	0.2838	0.1195	0.0177 *	1			
SH_+Ang_tRNA	0.7637	0.5376	0.0954	0.0117 *	0.7274	1		
MZ_+Ang_tRF	0.3631	0.0328 *	0.5028	0.0955	0.3049	0.1937	1	
SH_+Ang_tRF	0.0224 *	0.0023 **	0.3157	0.8034	0.0245 *	0.0135 *	0.0864	1

Table S4A : Significant differences estimation for m7G without cell line distinction.

	-Ang_tRNA	-Ang_tRF	+Ang_tRNA	+Ang_tRF
-Ang_tRNA	1			
-Ang_tRF	0.6476	1		
+Ang_tRNA	0.2239	0.0805	1	
+Ang_tRF	0.8886	0.53	0.2286	1

Table S4B : Significant differences estimation for m7G with cell line distinction.

	MZ_-Ang_tRNA	SH_-Ang_tRNA	MZ_-Ang_tRF	SH_-Ang_tRF	MZ_+Ang_tRNA	SH_+Ang_tRNA	MZ_+Ang_tRF	SH_+Ang_tRF
MZ_-Ang_tRNA	1							
SH_-Ang_tRNA	0.0106 *	1						
MZ_-Ang_tRF	0.0987	0.3057	1					
SH_-Ang_tRF	0.1139	0.3753	0.9729	1				
MZ_+Ang_tRNA	0.8119	0.0131 *	0.0789	0.1309	1			
SH_+Ang_tRNA	0.3571	0.0987	0.4134	0.4536	0.2181	1		
MZ_+Ang_tRF	0.4134	0.0987	0.4958	0.4332	0.3223	1	1	
SH_+Ang_tRF	0.1401	0.2897	0.9729	0.8384	0.0851	0.5177	0.5177	1

Table S5A : Significant differences estimation for m3C without cell line distinction.

	-Ang_tRNA	-Ang_tRF	+Ang_tRNA	+Ang_tRF
-Ang_tRNA	1			
-Ang_tRF	0.0276 *	1		
+Ang_tRNA	0.7901	0.0121 *	1	
+Ang_tRF	0.1011	0.5641	0.11	1

Table S5B : Significant differences estimation for m3C with cell line distinction.

	MZ_-Ang_tRNA	SH_-Ang_tRNA	MZ_-Ang_tRF	SH_-Ang_tRF	MZ_+Ang_tRNA	SH_+Ang_tRNA	MZ_+Ang_tRF	SH_+Ang_tRF
MZ_-Ang_tRNA	1							
SH_-Ang_tRNA	0.0293 *	1						
MZ_-Ang_tRF	0.6052	0.007 **	1					
SH_-Ang_tRF	0.8518	0.0207 *	0.8785	1				
MZ_+Ang_tRNA	0.2824	0.5054	0.065	0.1304	1			
SH_+Ang_tRNA	0.2284	0.6454	0.0499 *	0.1605	0.8785	1		
MZ_+Ang_tRF	0.8518	0.065	0.5737	0.7984	0.3823	0.2786	1	
SH_+Ang_tRF	0.9497	0.0281 *	0.6454	0.8785	0.2786	0.2345	0.9591	1

Table S6A : Significant differences estimation for D without cell line distinction.

	-Ang_tRNA	-Ang_tRF	+Ang_tRNA	+Ang_tRF
-Ang_tRNA	1			
-Ang_tRF	0.4496	1		
+Ang_tRNA	0.918	0.5311	1	
+Ang_tRF	0.5257	0.928	0.6514	1

Table S6B : Significant differences estimation for D with cell line distinction.

	MZ_-Ang_tRNA	SH_-Ang_tRNA	MZ_-Ang_tRF	SH_-Ang_tRF	MZ_+Ang_tRNA	SH_+Ang_tRNA	MZ_+Ang_tRF	SH_+Ang_tRF
MZ_-Ang_tRNA	1							
SH_-Ang_tRNA	0.9109	1						
MZ_-Ang_tRF	0.5193	0.5059	1					
SH_-Ang_tRF	0.6099	0.7624	0.7822	1				
MZ_+Ang_tRNA	0.9002	0.9106	0.6595	0.8089	1			
SH_+Ang_tRNA	0.918	0.9586	0.4787	0.7167	0.829	1		
MZ_+Ang_tRF	0.5847	0.5979	0.8628	0.8357	0.7688	0.6345	1	
SH_+Ang_tRF	0.7037	0.746	0.7889	0.9862	0.8833	0.7297	0.9037	1

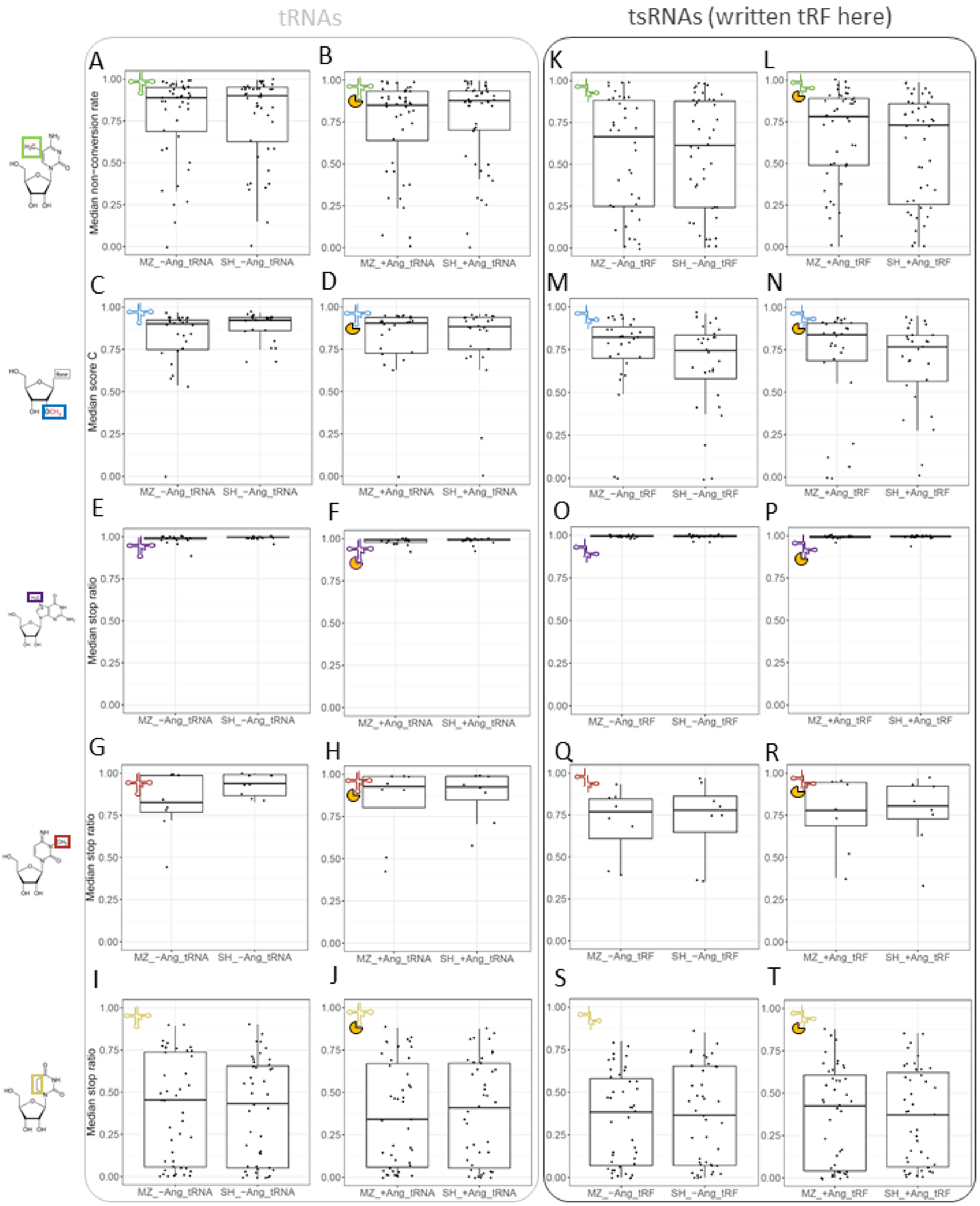

Figure S5 : Alternative representation of figure 5

In contrast to Figure 5 separating samples according to their treatment, Panels (A-J) correspond to intact tRNAs samples, while panels (K-T) show the results for tsRNAs samples. Modification levels for m^5C (A, B, K, L), Nm (2'-O-Me) (C, D, M, N), m^7G (E, F, O, P), m^3C (G, H, Q, R) and dihydrouridine D (I, J, S, T) are shown. The values shown here are modification level medians of the respective detection scores obtained for three biological replicates.

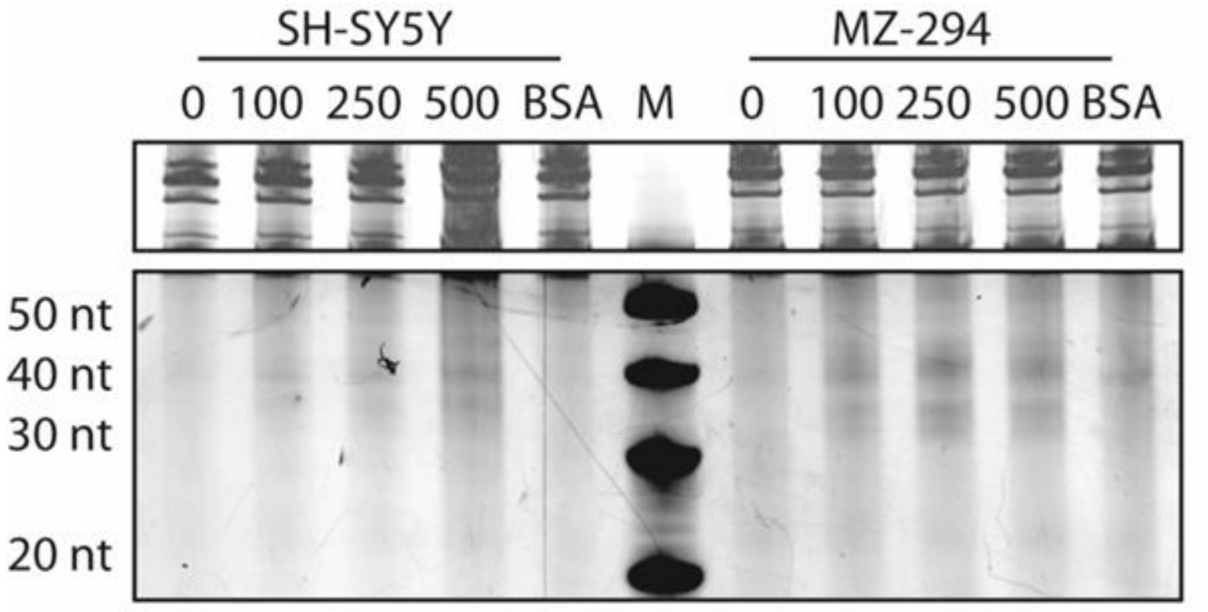

Figure S6: RNA gel showing RNA fragmentation in SH-SY5Y and MZ-294 cells treated with recombinant human Angiogenin. Cells were treated with recombinant human Angiogenin or BSA in Serum free Neurobasal media for 3 hours, and total RNA was loaded on a 15 % polyacrylamide TBE-UREA gel which was stained with SYBR Gold for visualisation. Execution and results of this experiments are in keeping with those previously published in [521] Hogg, M.C., et al., *5'ValCAC tRNA fragment generated as part of a protective angiogenin response provides prognostic value in amyotrophic lateral sclerosis.* Brain Commun, 2020. **2**(2): p. fcaa138.

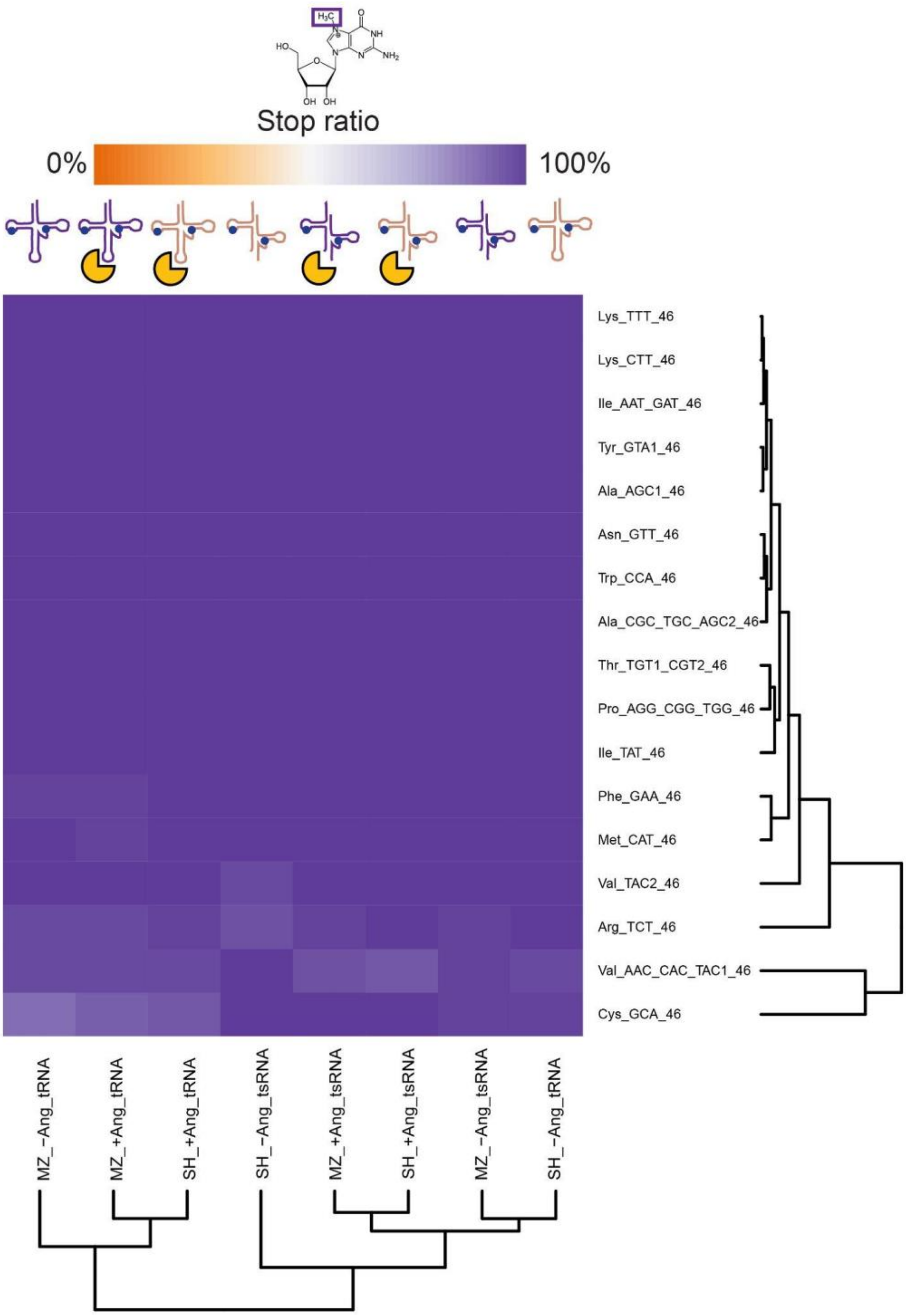

Figure S7 : Heatmap for the assessment of modification changes in individual isoacceptors and isodecoders of m7G sites.

This heatmap displays m7G stoichiometry across the different samples (X-axis) and the different sites retained for analysis (Y-axis) through its AlkAnilineSeq quantitative score stop ratio. This stoichiometry is color-coded from orange for low modification level through white for medium one to finish by purple for high. Each sample and each site are hierarchically clustered based on the Euclidean distances between each subset, represented by dendrograms at the bottom for samples and on the right for sites. No specific sites categorisation have been deduced from this heatmap.

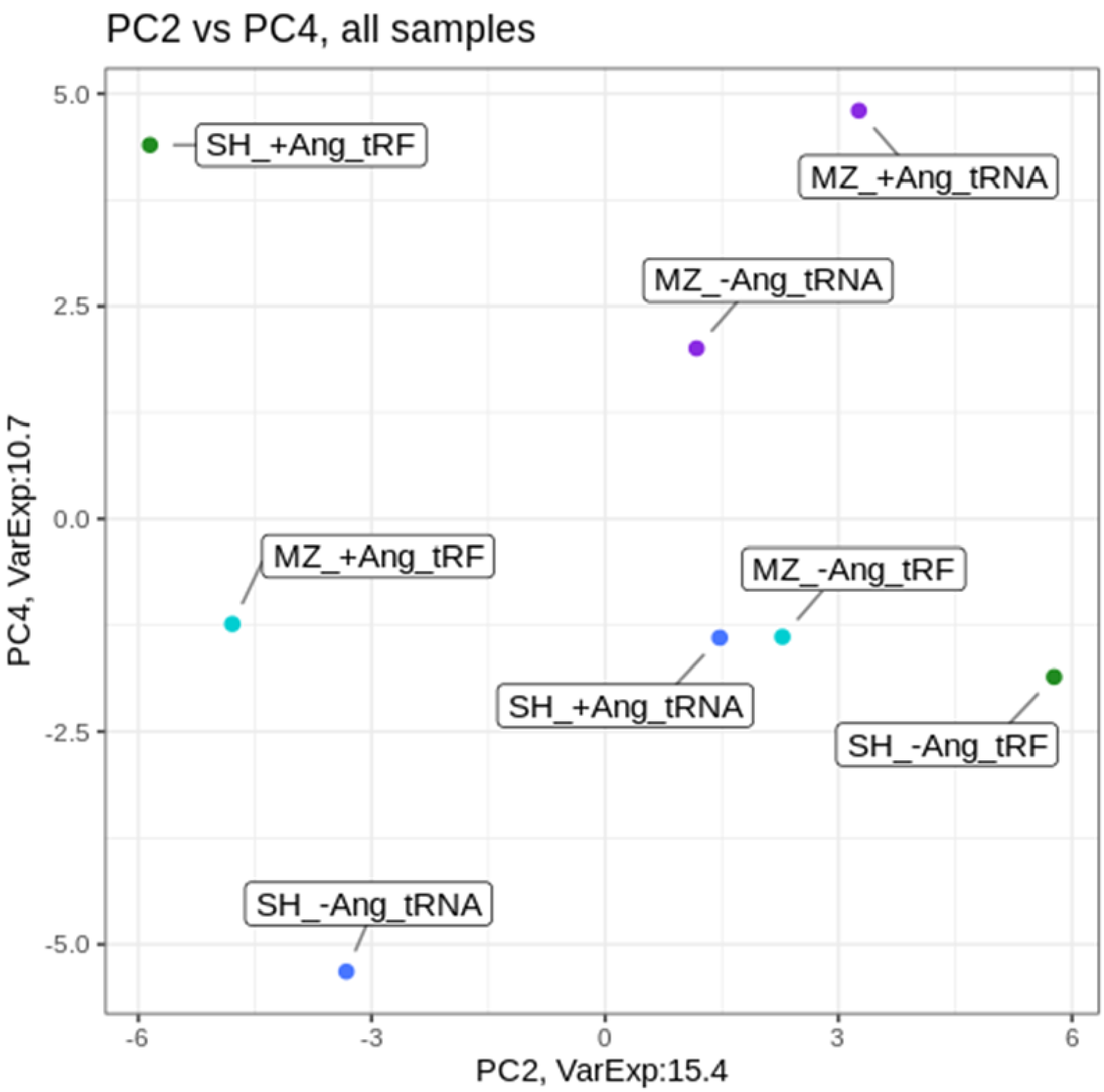

Figure S8 : Plot of PC2 versus PC4 segregates +ANG samples.

The combination of orthogonal vectors PC2 and PC4 from Figure 9 allows to illustrate a clear separation for tRNAs along the diagonal from bottom-left to top-right, while providing a distinct angiogenin clustering effect for tRFs along the X-axis. Correlating these two axes with the different sites provides the sites which best explains the biological differences for tRNAs and angiogenin effect for tsRNAs (here labeled “tRFs”) respectively.

Table S7. Contributors to the 4 first PC listed as loadings vectors. The latter correspond as correlation coefficients since PCA has been done on the data's correlation matrix.

Site	PC1 loadings	PC2 loadings	PC3 loadings	PC4 loadings	Modification
Ala_AGC1_17	0.41	0.17	-0.15	0.69	D
Ala_AGC1_20	0.51	0.20	-0.73	-0.04	D
Ala_AGC1_39	-0.92	-0.05	-0.20	-0.07	Nm
Ala_CGC_TGC_AGC2_49	-0.36	-0.11	0.28	0.74	m5C
Arg_ACG_16	-0.90	0.38	-0.09	0.00	D
Arg_ACG_47	0.01	-0.12	0.37	-0.36	D
Arg_CCG1_TCG_32	-0.42	-0.81	0.39	-0.02	Nm
Arg_CCT_CCG2_16	-0.50	0.08	0.76	0.02	D
Arg_CCT_CCG2_20	0.55	-0.53	0.07	0.17	D
Arg_CCT_CCG2_32	-0.84	0.33	0.29	0.11	m3C
Arg_TCT_16	-0.87	0.07	0.42	0.09	D

Arg_TCT_20a	-0.71	0.30	0.37	-0.51	D
Asn_GTT_16	0.53	0.67	-0.24	-0.12	D
Asn_GTT_20	0.33	0.40	0.81	-0.03	D
Asn_GTT_47	0.70	0.12	0.26	-0.57	D
Asn_GTT_54	-0.95	-0.13	-0.24	-0.09	Nm
Asp_GTC_20	0.76	-0.32	-0.11	-0.11	D
Asp_GTC_38	-0.21	0.66	0.14	-0.03	m5C
Asp_GTC_48	0.42	0.22	0.35	-0.63	m5C
Asp_GTC_49	0.08	0.38	-0.35	-0.80	m5C
Cys_GCA_20	-0.44	0.20	0.47	-0.18	D
Cys_GCA_32	-0.39	0.19	-0.61	-0.08	Nm
Cys_GCA_49	0.10	-0.62	0.52	0.43	m5C
Gln_CTG1_TTG_17	-0.27	0.89	0.09	0.08	D
Gln_CTG1_TTG_18	-0.84	-0.47	-0.12	-0.08	Nm

Gln_CTG1_TTG_20	-0.58	-0.45	0.18	-0.57	D
Gln_CTG1_TTG_20a	-0.76	-0.45	0.25	0.33	D
Gln_CTG1_TTG_32	0.37	-0.31	0.45	-0.66	Nm
Glu_CTC_TTC1_20	-0.72	0.34	0.27	0.38	D
Glu_CTC_TTC1_20a	-0.61	0.09	0.66	-0.39	D
Glu_CTC_TTC1_49	0.48	-0.43	0.33	-0.66	m5C
Glu_CTC_TTC1_50	0.66	-0.34	0.23	-0.60	m5C
Glu_CTC_TTC1_54	-0.80	-0.10	-0.25	-0.23	Nm
Glu_TTC2_20	-0.69	-0.66	0.21	0.08	D
Glu_TTC2_20a	-0.42	-0.83	-0.23	-0.13	D
Glu_TTC2_49	-0.55	0.18	-0.33	-0.67	m5C
Glu_TTC2_50	-0.65	0.24	-0.27	-0.51	m5C
Glu_TTC2_54	-0.96	-0.08	-0.19	-0.17	Nm
Gly_CCC1_GCC_38	0.78	-0.19	0.12	0.23	m5C

Gly_CCC1_GCC_48	0.71	-0.08	-0.32	-0.56	m5C
Gly_CCC1_GCC_49	-0.82	-0.21	-0.21	-0.34	m5C
Gly_CCC1_GCC_50	0.14	-0.60	-0.03	-0.58	m5C
Gly_CCC2_20	-0.89	-0.27	0.14	0.06	D
Gly_CCC2_32	0.56	-0.04	0.81	0.01	Nm
Gly_CCC2_39	-0.55	0.65	-0.06	-0.46	Nm
Gly_CCC2_49	0.57	-0.46	0.39	-0.54	m5C
Gly_CCC2_50	0.72	-0.50	0.15	-0.45	m5C
Gly_TCC_20	-0.74	-0.54	0.00	0.31	D
Gly_TCC_49	0.10	0.69	-0.35	0.47	m5C
Gly_TCC_50	0.56	-0.19	-0.20	-0.69	m5C
His_GTG_17	0.42	-0.23	-0.73	0.09	D
His_GTG_20	0.66	-0.55	-0.05	-0.34	D
His_GTG_20a	0.68	-0.33	0.36	-0.51	D

His_GTG_49	-0.85	-0.47	0.17	-0.06	m5C
His_GTG_50	0.08	-0.47	-0.24	-0.14	m5C
Ile_AAT_GAT_48	-0.96	0.03	0.27	0.05	m5C
Leu_CAG_CAA_32	-0.94	0.02	-0.24	-0.24	Nm
Leu_CAG_CAA_44	-0.81	-0.25	-0.49	0.06	Nm
Leu_CAG_CAA_48	-0.98	-0.02	0.07	-0.09	m5C
Leu_CAG_CAA_e2	0.48	-0.27	0.18	-0.60	m3C
Leu_TAA_48	-0.94	0.17	0.11	-0.03	m5C
Lys_CTT_16	-0.35	0.24	0.32	0.67	D
Lys_CTT_48	-0.97	-0.12	0.16	0.04	m5C
Lys_CTT_54	-0.86	0.15	-0.08	-0.31	Nm
Lys_TTT_16	-0.75	0.57	-0.17	0.12	D
Lys_TTT_49	-0.98	0.14	0.05	0.06	m5C
Lys_TTT_54	-0.80	0.39	-0.14	-0.19	Nm

Met_CAT_20	0.53	0.23	0.13	-0.65	m3C
Met_CAT_34	-0.38	0.56	0.22	-0.10	Nm
Met_CAT_47	-0.24	0.77	-0.10	-0.27	D
Met_CAT_49	-0.84	-0.01	0.17	-0.24	m5C
Phe_GAA_16	-0.80	-0.08	0.27	-0.38	D
Phe_GAA_17	-0.78	-0.03	0.36	-0.13	D
Phe_GAA_34	-0.86	-0.06	-0.18	0.12	Nm
Phe_GAA_47	0.22	0.46	0.80	-0.03	D
Phe_GAA_49	-0.94	-0.08	0.11	-0.28	m5C
Pro_AGG_CGG_TGG_20	-0.33	-0.86	-0.07	0.03	D
Pro_AGG_CGG_TGG_32	-0.89	-0.07	-0.41	-0.16	Nm
Pro_AGG_CGG_TGG_49	-0.74	-0.45	-0.40	-0.12	m5C
Pro_AGG_CGG_TGG_50	-0.89	-0.20	-0.25	-0.24	m5C
Ser_AGA_TGA_17	0.33	-0.65	0.34	0.36	D

Ser_AGA_TGA_18	-0.85	-0.15	-0.49	-0.05	Nm
Ser_AGA_TGA_20	0.22	0.60	0.41	-0.32	D
Ser_AGA_TGA_20a	0.42	-0.57	0.30	0.51	D
Ser_AGA_TGA_32	-0.86	-0.45	-0.14	0.18	m3C
Ser_AGA_TGA_44	-0.84	-0.24	-0.41	0.06	Nm
Ser_AGA_TGA_49	-0.98	0.10	0.09	-0.02	m5C
Ser_GCT_18	-0.79	0.32	-0.14	-0.38	Nm
Ser_GCT_32	-0.77	-0.47	0.14	0.20	m3C
Ser_GCT_39	-0.37	-0.44	-0.76	-0.22	Nm
Ser_GCT_44	-0.81	-0.35	-0.42	0.09	Nm
Ser_GCT_49	-0.94	0.18	0.09	0.02	m5C
Thr_AGT_CGT3_TGT2_32	-0.85	0.33	0.30	-0.25	m3C
Thr_AGT_CGT3_TGT2_47	0.04	-0.90	0.23	-0.10	D

Thr_CGT1_32	-0.76	-0.48	0.34	0.25	m3C
Thr_CGT1_48	-0.99	-0.09	0.07	0.02	m5C
Thr_CGT1_72	-0.81	-0.37	0.45	0.06	m5C
Thr_TGT1_CGT2_20	0.72	0.23	-0.38	-0.14	D
Thr_TGT1_CGT2_32	-0.95	0.16	0.06	-0.06	m3C
Thr_TGT1_CGT2_48	-0.95	-0.23	0.07	0.00	m5C
Thr_TGT1_CGT2_72	-0.71	-0.27	0.34	0.08	m5C
Trp_CCA_39	-0.91	0.29	0.02	-0.13	Nm
Tyr_GTA1_16	0.90	-0.10	-0.13	0.02	D
Tyr_GTA1_17	0.98	0.05	-0.15	0.07	D
Tyr_GTA1_47	-0.76	0.43	0.04	-0.01	D
Tyr_GTA1_48	-0.97	0.09	0.12	0.04	m5C
Val_AAC_CAC_TAC1_17	0.68	-0.37	0.43	0.42	D
Val_AAC_CAC_TAC1_38	0.42	0.42	0.72	-0.14	m5C

Val_TAC2_20	-0.55	0.65	0.38	0.27	D
Val_TAC2_20a	0.67	0.41	0.36	0.17	D
Val_TAC2_48	-0.72	-0.02	0.52	-0.06	m5C
Val_TAC2_49	-0.68	-0.18	0.52	-0.30	m5C

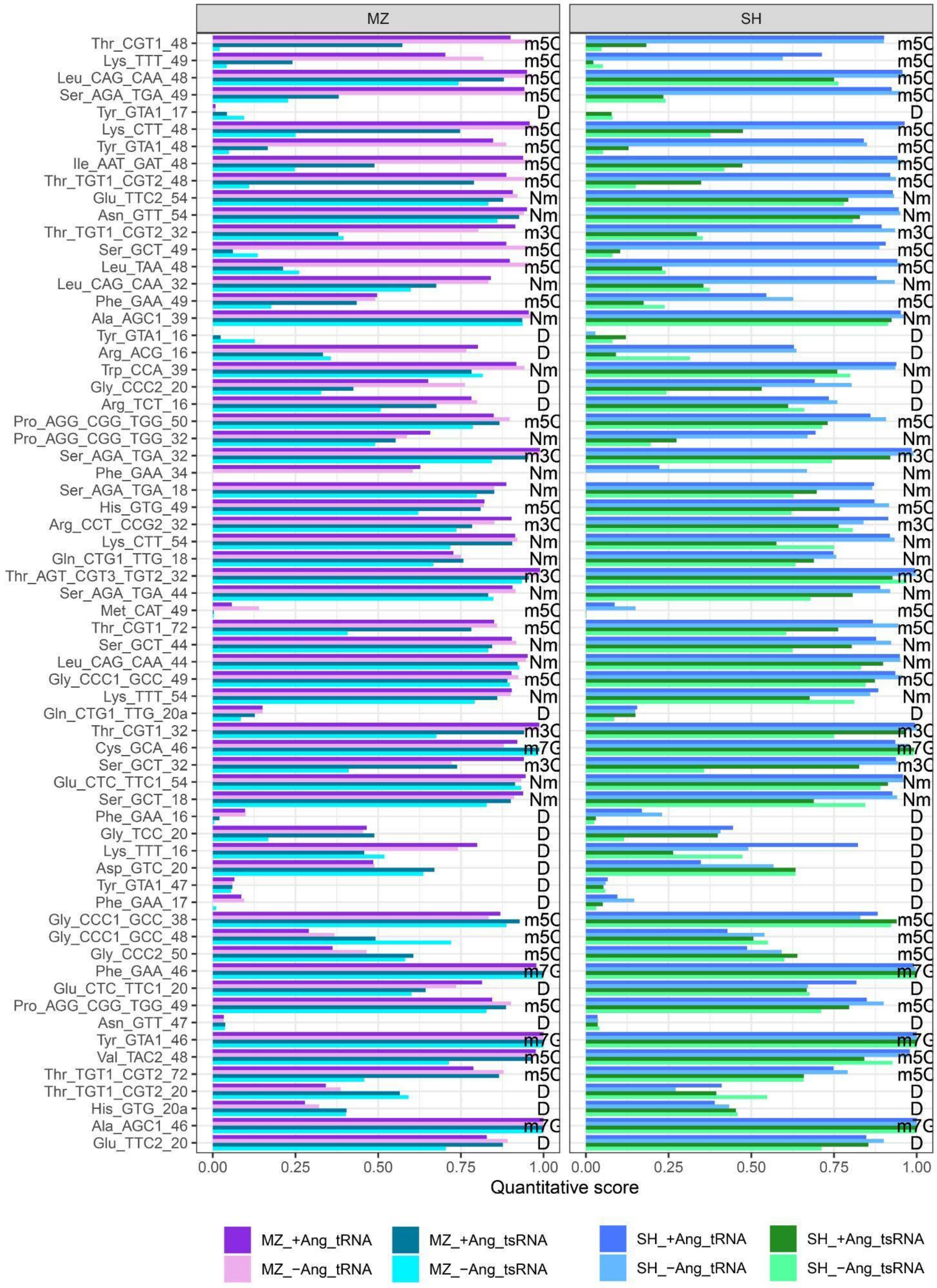

Figure S9: Barplots displaying modification stoichiometry values for main contributors of PC1.

Left panels for MZ cells, right panels for SH cells. The nature of modified nucleotide is shown at the top of each bar, and the identity of tRNA/tsRNA site on the left along the Y-axis. Color code indicates the identity of the sample (tRNA/tsRNA) and treatment with ANG (-Ang/+Ang).
